# Microplastics, Polycyclic Aromatic Hydrocarbons, and Heavy Metals in Milk: Analyses and Induced Health Risk Assessment

**DOI:** 10.3390/foods13193069

**Published:** 2024-09-26

**Authors:** Andreea Laura Banica, Cristiana Radulescu, Ioana Daniela Dulama, Ioan Alin Bucurica, Raluca Maria Stirbescu, Sorina Geanina Stanescu

**Affiliations:** 1 Institute of Multidisciplinary Research for Science and Technology, Valahia University of Targoviste, 13 Sinaia Alley, 130004 Targoviste, Romania; banica.andreea@icstm.ro (A.L.B.); dulama.ioana@icstm.ro (I.D.D.); bucurica_alin@icstm.ro (I.A.B.); stirbescu.raluca@icstm.ro (R.M.S.); geanina.stanescu@icstm.ro (S.G.S.); 2 Doctoral School Chemical Engineering and Biotechnology, National University of Science and Technology Politehnica of Bucharest, 313 Splaiul Independenței, 060042 Bucharest, Romania; 3 Faculty of Sciences and Arts, Valahia University of Targoviste, 13 Sinaia Alley, 130004 Targoviste, Romania; 4 Academy of Romanian Scientists, 3 Ilfov, 050044 Bucharest, Romania

**Keywords:** microplastics, PAHs, heavy metals, milk, micro-FTIR, HPLC-FLD, ICP-MS, health risk

## Abstract

The current study aims to develop isolation protocols for several contaminants of emerging concern (i.e., microplastics (MPs), polycyclic aromatic hydrocarbons (PAHs), and heavy metals (HMs)) from different commercial brands and raw milk samples and also to quantify and characterize the risks of these contaminants pose to human health. The quantification, shape, color, and chemical composition of MPs were achieved using optical microscopy, micro-Fourier transform infrared spectroscopy, and scanning electron microscopy coupled with energy-dispersive spectroscopy. Based on the MP dimensions highlighted by the aforementioned techniques, it can be stated that their length ranges between tens of micrometers and a few centimeters; plus, the thickness in some cases reaches more than 15 µm, and the structure of the MPs can be mostly described as a fibriform with a glossy/matte aspect. The polymeric structures identified were polyamides, poly(methyl methacrylate), polyurethane, polyester, and polyethylene. Chemical investigations (PAHs and HMs concentrations) were performed by high-performance liquid chromatography with fluorescence detection and inductively coupled plasma mass spectrometry, respectively. The pollution load index (1.091–7.676) and daily intake of MPs for adults (0.021–1.061 n·kg^−1^·d^−1^) and children (0.089–4.420 n·kg^−1^·d^−1^) were calculated. It can be concluded that the presence of MPs in milk supports the hypothesis that microplastics can act as carriers for other contaminants (HMs and PAHs), thus increasing the threat to health.

## 1. Introduction

Contaminants of emerging concern or emerging contaminants (ECs) are those chemical compounds that are not currently or have been only recently stipulated in legislation and about which concerns exist regarding their impact on the environment and human health in terms of possible interaction mechanisms, distribution, and migration pathways. In this respect, the latest research has shown that concerns about the harmful effects of emerging contaminants on ecosystems and human health have grown [1,2]. Such worldwide concern is understandable, because a series of ECs, such as microplastics (MPs) [3,4], heavy metals [5,6], disinfection by-products [7], pharmaceutical and personal care products (PPCPs) [8], and persistent organic compounds (e.g., pesticides, polycyclic aromatic hydrocarbons (PAHs), etc.) [9], which are mainly non-biodegradable, have quickly polluted the marine, freshwater, and terrestrial ecosystems as well as drinking water and the food chain.

Briefly, microplastics are solid fragments of extremely small sizes (<5 mm), usually fibers, that mainly contain different polymers (e.g., polyethylene (PE), polystyrene (PS), polypropylene (PP), polyethylene terephthalate (PET), and nylon) [10]. There is great interest among researchers in the capacity of MPs to transport highly toxic metals and other non-degradable contaminants, such as PAHs. Polycyclic aromatic hydrocarbons are mainly generated from pyrolysis and/or an incomplete combustion of organic matter [11]. They have been classified as genotoxic and possibly/probably carcinogenic to the human body along with heavy metals. Among these, benzo(a)pyrene is the most studied, being included in the first group of human carcinogens [12]. Benzo(a)anthracene, chrysene, and benzo(b)fluoranthene are also compounds found in food products with a high carcinogenic risk [12]. Among all the basic foods consumed by people from the youngest ages to the elderly, milk is the most complex and most used food. Milk is considered the most complete food due to its complex chemical composition, which includes fat substances like glycerides, phosphatides, and steroids; non-fat substances like caseins and steric proteins, lactose, and minerals (Ca, Se, Zn, Mg, and I); and other substances like enzymes, vitamins, pigments, and gases. Starting from this brief description of the chemical composition of milk, one can see why the investigations regarding the presence of emerging contaminants such as MPs, PAHs, or heavy metals are still limited.

The current research proposes a protocol to isolate MPs from milk and discusses a comprehensive physicochemical investigation of emerging contaminants, including MPs, PAHs, and heavy metals, that are potentially present in milk, simultaneously assessing the health risks linked with human exposure to them. At the beginning of this scientific incursion, one question came up: “Why are there only a few studies regarding the isolation of microplastics from milk?” The answer is, unlike other cases such as water, fish, fruits, vegetables, plants, drinks (juices, energy drinks, or beer), and even meat, milk is an extremely complex food, as mentioned above.

First of all, the isolation of MPs from milk samples containing fatty (saturated or unsaturated acids) and non-fatty (proteins, amino acids, vitamins, pigments, and minerals) organic matrices is extremely laborious in terms of finding the most effective isolation methods, especially if the milk has different fat concentrations (1.5–1.8% or 3.5–3.8%) or is processed and packaged differently (bio or non-bio and intended for adults or children). Taking into account these complex organic matrices of milk, the authors proposed for the first time a benchmark isolation protocol for MPs from different branded/raw milk (subject of patent application [13,14]) as well as from raw milk smallholder farmers. This work gives the scientific community the opportunity to advance this cause–effect research and gain new insights into human safety. Second, the morphological and chemical investigation of isolated MPs achieved by this research supports, for the first time, the importance of the correlation between the material’s chemical composition, structure, size, color, and source of origin, with complementary techniques, like micro-Fourier transform infrared spectroscopy (micro-FTIR), and with known ones, like optical microscopy (OM) and scanning electron microscopy coupled with energy-dispersive spectroscopy (SEM-EDS), contributing to the development of a database related to MPs in foods, particularly in milk. Last but not least, this study is the first of its kind to demonstrate that the presence of MPs in milk, regardless of whether it is brand, commercial, or from small producers, is corroborated with the presence of other emerging contaminants, such as HMs and PAHs, which can represent a risk for human health, especially for children aged between 0 and 10 years, according to data related to pollution load index (PLI) and estimated daily intake (EDI). However, in addition to assessing the health risks associated with the heavy metals found in milk, the chosen samples were also analyzed to ascertain the values of the estimated daily intake (EDI) and health risk index (HRI) with the goal of determining the riskiness of their consumption with regard to human health.

## 2. Materials and Methods

### 2.1. Materials and Reagents

The reagents used in performing the analyses (liquid chromatography, inductively coupled plasma mass spectrometry) were of HPLC grade (Merck, KGaA, Darmstadt, Germany, and Sigma-Aldrich, Saint Louis, MO, USA). To avoid accidental contamination of the samples (including ultrapure water) or possible interferences in chemical investigations, the liquid ones were filtered. All materials used for further investigations were properly cleaned and sterilized likewise at a temperature of 100 °C for 48 h in a forced convection Venticell^®^ oven (BMT Medical Technology, Brno, Czech Republic). Filtration of the milk samples was performed using filter paper with 12–15 µm porosity, VWR^®^ Grade 413 (VWR International, Radnor, PA, USA). Furthermore, all filters and reagents were checked step by step. First, three random filters were verified using optical microscopy and showed no MP traces or any materials/other fibers. In the second step, three new random filters were used separately to filter the reagent (H_2_O_2_); these filters were the subjects of optical microscopy investigations and again showed no traces of contamination as the filters were unaltered. The main importance of this activity was to ensure that any source (justified or unjustified) of contamination was ruled out throughout the process before moving onto milk filtration.

### 2.2. Sampling and Sample Preparation

The selection of milk samples was based on different characteristics such as the quality marketed by the producers (conventional or organic milk), popularity of the brand, scaling sales, type of packaging, and price of the product. A total of nine brands having different fat content (between 1.5 and 3.8%) were chosen and purchased from Romanian markets. Regarding the milk packaging, it can be specified that the materials used in this process are from the following categories: Tetra Pack, plastic, or glass. After sampling, the branded milk was divided in conventional milk and organic milk as shown in Tables S1 and S2. Furthermore, four extra samples of raw milk (coded LF1, LF2, LF3, and LF4) were collected and transported in glass vessels (cleaned and sterilized) using freezers to the laboratory; their provenance was assigned to some small Romanian farms. Considering the contamination risk that may occur, it was decided that all samples preparation (including the sampling stage) was to be performed under strict regulation of the laboratory environment where no kind of airborne particles may interfere. Therefore, these activities were carried out in a cleanroom (according to ISO 14644-1:2015, Class 1000-ISO6) [15], and non-textile lab coats (i.e., cotton) and particle-free nitrile gloves were worn the entire time, while the sleeves of the lab coats were in advance inserted and secured inside the gloves. The glass vessels were sequentially rinsed at least three times with anhydrous ethanol and ultrapure water. Samples were carefully handled and covered with aluminum foil until the isolation process. The isolation protocol of MPs was repeated three times for each sample, and the data were obtained by averaging.

Depending on the monitored emerging contaminants, we used different methods of preparing milk samples. Thus, for the quantification and characterization of microplastics, a unique and innovative isolation method was used (which is the subject of two patent applications [13,14]). The preparation of milk samples to determine the PAHs followed a slightly modified method presented by Girelli et al. [16]. To determine the concentration of heavy metals, the classical method of digestion under pressure was used, the goal being the total degradation of the inorganic matrix, to obtain the ionic species that were analyzed by inductively coupled plasma mass spectrometry (ICP-MS).

#### 2.2.1. Isolation Procedure of Microplastics 

The isolation procedure of MPs (subject of two application patents (i) national no. RO137927A0/26.09.2023 [13] and (ii) international PCT/RO2024/000010/15.04.2024 [14]), briefly, consists of the following: (1) standard amount of milk purchased from the market and analyzed was 3 L for each of the samples (analysis in triplicate). From each liter of milk, 100 mL was divided into 200 mL Erlenmeyer beakers to make the handling and processing of the samples more efficient. One of the following reagents was added to each subsample: 5 mL NaOH (40%), 0.4 g NaOH (~100%), 10 mL H_2_O_2_ (30%), or 10 mL petroleum ether. (2) After adding the reagents, the milk samples were placed on the IKA^®^ RT 5 shaker (IKA) and maintained for 10 min at 150 rpm. It was mentioned that in the first tests, different time intervals (i.e., 10, 30, and 60 min) and different rotation speeds (i.e., 50, 100, and 150 rpm) were used (Appendix A). Since the purpose of this step is to homogenize the reagent in the milk sample, as well as optimize the working time, the parameters mentioned above (10 min at 150 rpm) are considered sufficient to achieve the proposed goal. (3) To obtain complete digestion of the milk samples, we used an ultrasonic bath, varying the temperature (room temperature, 30 °C, 45 °C, 60 °C, and 75 °C, respectively) and the time (20, 30, 60, and 90 min, respectively) (Appendix B). This stage consisted of ultrasonication in the ultrasonic bath type VWR^®^ Ultrasonic Cleaner USC—TH (VWR International LLC) for which the optimal temperature parameter was established at 30 °C for 20 min. At temperatures below 30 °C, milk fat globules rise to the surface and are not digested or filtered. Furthermore, in tests performed at higher temperatures, we observed no significant differences compared to the established optimal temperature of 30 °C. The temporal parameter showed, on the other hand, that a shorter ultrasonication time results in an incomplete digestion of the samples and implicitly the impossibility of using them in the filtration process. After the ultrasonication was finished, the milk samples were kept on a water bath at a constant temperature of 60 °C until the time of total filtration. (4) One of the effective approaches for the isolation of MPs from food matrices is membrane-based separation. The simple method uses differential pressure to favor membrane filtration and does not require a complicated setup. The application of pressure allows the retention of microplastics on the surface of various filters. Filtration was the last stage of the work protocol to isolate microplastics from milk samples and was achieved using a 3-station stainless-steel filter manifold (Labbox Labware, Barcelona, Spain), a vacuum pump with a flow rate of 18 L/min, and 5–13 µm porosity filters obtained from high-purity cellulose. (5) After performing the preliminary tests, the optimal procedure for isolating MPs from milk samples was established as follows: 1 L of milk was distributed in two Erlenmeyer flasks of 500 mL each, over which 50 mL of H_2_O_2_ (30%) was added. The obtained sample was homogenized on the magnetic stirrer (10 min at 150 rpm), ultrasonicated for 20 min at 30 °C, and filtered on a cellulose membrane with a porosity of 12–15 µm.

#### 2.2.2. Preparation of Milk Sample to Determine Polycyclic Aromatic Hydrocarbons by High-Performance Liquid Chromatography with Fluorescence Detection

In a polypropylene test tube equipped with a threaded stopper, a 2 mL sample (conventional/organic milk) was subjected to saponification with 0.4 M sodium hydroxide (HPLC purity, Merck, Darmstadt, Germany) in ethanolic solution (HPLC grade, Merck, Germany). The obtained mixture was heated to 60 °C, kept in a water bath for 30 min, and cooled to room temperature. On the samples thus obtained, 2 mL of n-hexane (HPLC grade, Merck, Germany) was added, and the resulting mixture was stirred for 1 min. The final samples obtained were centrifuged for 3 min at 4000 rpm. The supernatant resulting from the centrifugation was recovered in another test tube; another 2 mL of n-hexane was added to the residue, and the method was repeated (stirring and centrifugation), completing the procedure with the recovery of the supernatant. The two supernatants were collected in the same tube, obtaining a final supernatant. The method applied to determine polycyclic aromatic hydrocarbons in milk was according to the description of Girelli et al. [16]. In this regard, the final supernatant was concentrated in a nitrogen stream (purity 99.999%) using the MutliVap 10 concentration system (LabTech, Sorisole, Italy) in 50 mL vials. The residue was recovered with 100 mL of 100% acetonitrile (HPLC grade, Merck, Germany) and filtered through a 0.45 µL PTFE membrane (Hypo, Bucharest, Romania). From the sample obtained, 5 µL was injected into the chromatographic column.

#### 2.2.3. Digestion of Milk Samples to Determine the Heavy Metals Concentration by Inductively Coupled Plasma Mass Spectrometry

To determine the content of heavy metals in milk, assisted chemical digestion was performed using a TOPWAVE Digestor (Analytik Jena, Jena, Germany). The samples were prepared in polytetrafluoroethylene (PTFE) tubes as follows: over 5 mL of the milk sample, 4 mL of nitric acid 65% (HPLC grade, Lach–Ner, Neratovice, Czechia), and 2 mL of hydrogen peroxide 30–35% (Sigma Aldrich, St. Louis, MO, USA) were added. Blank samples were prepared, using the previously mentioned method, by replacing the milk sample with ultrapure distilled water. The obtained samples were kept at rest for 30–40 min, at room temperature, to destroy the organic matrix. Finally, chemical digestion was performed under pressure and temperature (Table 1) to destroy the inorganic matrix. After completion of the digestion program, the samples were removed from the digester and allowed to cool to room temperature. After cooling the samples, the PTFE tubes were opened, and the sample was transferred into 25 mL volumetric flasks and made up to the mark with distilled water. The sample preparation procedure was achieved according to the method developed by Kilic-Altun and Aydemir [17].

### 2.3. Analytical Techniques

#### 2.3.1. Optical Microscopy

The microplastics isolated on filters were identified using optical microscopy (OM). For this purpose, the magnification factors ranged from 6.5× to 100×, depending on the complexity or the size of the observed particles to highlight certain aspects of interest related to them. In this sense, the Primo Star and Stemi 2000-c optical microscopes (Carl Zeiss Microscopy GmbH, Jena, Germany) were used for complementary reasons, allowing the examination of the samples in both transmitted and reflected light. The resulting images were acquired using ZEN 2012 (blue edition) (Version 1.1.2.0) and an Axiocam 105 digital video camera (Carl Zeiss Microscopy GmbH, Jena, Germany). Optical microscopy helped not only for the identification and physical characterization of microplastics but also for their isolation.

#### 2.3.2. Micro-Fourier Transform Infrared Spectroscopy

Fourier-transform infrared spectroscopy (FTIR) is the most common vibrational technique used to assess molecular motion and fingerprinting species based on the inelastic scattering of a monochromatic excitation source using an energy range from 200 to 8000 cm^−1^. Micro-Fourier transform infrared spectroscopy (micro-FTIR) is an important non-destructive and non-invasive tool that provides specific information about chemical bonding and molecular structure by using both an analysis of small chemical differences in samples (i.e., chemical area/line mapping) and FTIR imaging (i.e., particle analysis and multi-spot testing) [3,8,13,14]. The next step of this research, the IR spectrum, was necessary to strengthen or dispel any possible doubt regarding the presence of any polymer link of a synthetic or semisynthetic nature in the microparticles (supposed to be microplastics) discovered after visual identification (i.e., OM, SEM) and the isolation stage, respectively. For this reason, the micro-FTIR technique was performed on isolated MPs using a Vertex 80v spectrometer, equipped with a diamond ATR crystal accessory, for a high refractive index bulk sample and with a Hyperion 3000 microscope (Bruker Optics GmbH & Co. KG, Ettlingen, Germany). A Hyperion microscope is characterized by a 600–7500 cm^−1^ spectral range, 0.2 cm^−1^ spectral resolution, and ±1 µm accuracy. The design allows for obtaining the highest performance for the visual examination and infrared analysis of any sample.

#### 2.3.3. Scanning Electron Microscopy—Energy-Dispersive X-ray Spectroscopy 

Scanning electron microscopy—energy-dispersive X-ray spectroscopy (SEM-EDS) is an analytical technique increasingly used that involves investigating different materials’ crystal morphology (i.e., metals, plastics, alloy, metallic coating interfaces, etc.), contaminant identification, particle size, composition distribution analysis, surface features analysis for micro-constituents or micro-inclusion content, identification of organic to metal bonding failure modes, and more. On one hand, SEM is the imaging part of the entire technique, and on the other, the EDS is the part that determines elemental concentrations. At last, the SU-70 electron microscope (Hitachi, Ibaraki, Japon) coupled with an UltraDry energy-dispersive X-ray spectrometer (Thermo Fisher Scientific, Waltham, MA, USA) was used to characterize morphologically as well as for the elemental composition of the isolated microplastics. The SU-70 SEM microscope is a field emission type (FE-SEM) that allows for obtaining high-resolution images (1 nm at the acceleration voltage of 15 kV) because it operates under high-pressure conditions (10^−8^ Pa). SEM images were obtained at an acceleration voltage of 1 kV and a working distance of approximately 16 mm. The elemental content was determined using an acceleration voltage of 15 kV and the Phi-Rho-Z background correction technique available in the NSS program (Version 3.0).

#### 2.3.4. High-Performance Liquid Chromatography with Fluorescence Detection

Determination of the 16 types of PAHs in milk samples was performed by high-performance liquid chromatography with fluorescence detection (HPLC-FLD) using a 1260 Infinity II chromatograph (Agilent, Santa Clara, CA, USA) equipped with two detectors: a diode array detector (DAD) and fluorescence detector (FLD). Both the diode array detector (DAD) and fluorescence detector (FLD) were tested for the qualitative and quantitative determination of PAHs in milk samples, but the FLD demonstrated a higher accuracy than the DAD. The automatic processing of the obtained data was carried out with OpenLab CDS 2.7 software (Agilent Technologies Inc., Santa Clara, CA, USA). For a good separation and identification of PAHs, a chromatographic column type P.N.A 7001100X046 (Agilent Technologies Inc., Santa Clara, CA, USA) was used (pore size 200 Å; product size 100 × 4.6 mm; particle size 3 μm). The gradient elution program using solvent A (water H_2_O) and solvent B (acetonitrile CH_3_CN) was programmed as follows: 40% B (0–22 min; 1.8 mL·min^−1^), 90% B (23 min; 1.8 mL·min^−1^) to 100% B linearly (29 min; 3.5 mL·min^–1^), and 40% B (29.5–34 min; 1.8 mL·min^−1^). 

The column temperature was set at 30 °C, and the limit of quantification (LOQ) values of the method for PAHs were 0.20 ng·mL^−1^ for naphthalene and acenaphthene; 0.02 ng·mL^−1^ for fluorene, anthracene, fluoranthene, pyrene, benzo[a]anthracene, chrysene, benzo[b]fluoranthene, benzo[k]fluoranthene, dibenzo[a,h]anthracene, benzo [g,h,i]perylene, indeno(1,2,3-cd)pyrene, and dibenzo[a,h]anthracene; 0.5 ng·mL^−1^ for acenaphthylene; and 0.01 ng·mL^−1^ for benzo[a]pyrene, respectively.

#### 2.3.5. Inductively Coupled Plasma Mass Spectrometry

The samples’ metal content (i.e., Cr, Mn, Cu, Ni, Zn, Sr, and Pb) was performed by ICP-MS using an iCAP™ Qc device (Thermo Fisher Scientific, Erlangen Germany). The measurements were achieved in triplicate in the standard mode (STD), using the Qtegra Intelligent Scientific Data Solution. The standard deviation (SD) values were less than 10%. The quantification of this technique was performed by a standard curve procedure. Metals’ calibration curves showed good linearity over the concentration range (0.1 to 10.0 mg·L^−1^) with R^2^ correlation coefficients in the range of 0.997 to 0.999. The analytical curves for each analyzed element were prepared using stock standard solutions (Merck, Germany). The limits of detection (LODs) and quantitation (LOQs) of analyzed elements were established using the calibration data. The precision of the ICP-MS method was performed using the standard material NIST 1869—Infant/Adult Nutritional Formula II. The standard and sample standard deviation, as well as the recovery rate, were calculated.

### 2.4. Data Analysis

#### 2.4.1. Pollution Load Index of Microplastics

The pollution load index (*PLI*) was described by Tomlinson et al. [18] as

(1) $$PLI=\sqrt{\frac{C_{i}}{C_{0}}}$$

where *C_i_* represents the MPs content (expressed as n·kg^−1^) determined in the milk sample; and *C*_0_ represents the minimum reported average MPs concentration in processed foods [19,20]—*C*_0_ = 1.68 n·kg^−1^. Lin et al. [20] established the hazard levels as a function of *PLI* values (Table 2).

#### 2.4.2. Estimated Daily Intake for MPs

The estimated daily intake (*EDI*) for MPs can be calculated using the equation below as described by Lin et al. [20]:

(2) $$EDI=\frac{C_{i}\cdot I_{r}\cdot E_{f}\cdot E_{d}}{B_{w}\cdot A_{t}}\left[n\cdot {kg}^{-1}\cdot d^{-1}\right]$$

where *C_i_* represents the MPs content (expressed as n·kg^−1^) determined in the milk sample; *I_r_* represents the ingestion rate (expressed as kg·d^−1^); *E_f_* represents the exposure frequency (expressed as d·y^−1^); *E_d_* represents the exposure duration (expressed as y); *B_w_* represents the body weight (expressed as kg); and *A_t_* represents the average exposure time (expressed as d). All the values of these parameters are presented in Table 3.

#### 2.4.3. Estimated Daily Intake of Heavy Metals

The estimated daily intake (*EDI*) can be obtained using the following equation [21,22]:

(3) $$EDI=\frac{C_{milk}\cdot I}{BW}$$

where *EDI* is expressed in mg·kg^−1^·day, *C_milk_* represents the metal concentration in milk and is expressed in mg·L^−1^; *I* is expressed in L·day^−1^: for children, *I* = 0.625 L·day^−1^; for adults, *I* = 0.750 L·day^−1^, *BW* represents the body weight and is expressed in kg: for children, *BW* = 14 kg and for adults *BW* = 70 kg.

#### 2.4.4. Health Risk Index

The health risk index (*HRI*) is the evaluation of the health risks induced by the consumption of milk. The *HRI* can be estimated as the ratio between the *EDI* and the reference oral dose (*R_f_D*) [22,23,24] (Table 4).

#### 2.4.5. Statistical Analysis

Cluster analysis plays an essential role in understanding and exploiting complex data, providing a powerful tool for segmenting, simplifying, and analyzing data in various domains [25]. Cluster analysis involves the grouping of similar objects or with similar content in order to reduce the data used to sort the cases, observations, or variables of a given data set into homogeneous groups that differ from each other [26]. At the same time, cluster analysis along with principal components analysis are among the most used tools to explore the similarities between samples, and they also contribute to the organization of data by eliminating redundancies and detecting anomalies or other objects that do not fit into the characteristics of the group [26]. The recorded data were analyzed using IBM SPSS Statistics software (SPSS Inc., Chicago, IL, USA, 2011). 

## 3. Results and Discussion

### 3.1. Isolation Procedure of Microplastics

Microplastic contamination of the food chain is receiving increased attention both in the public environment and in the scientific community. Since the specialized literature does not have many studies applied to microplastics identified in milk and dairy products, except for the one published by Kutralam-Muniasamy et al. [27], the protocols presented in this study are characterized by a high degree of novelty and originality. In their study, Kutralam-Muniasamy et al. [27] visually identified the microplastics contained in 23 milk samples (whole milk, lactose-free milk, and baby formulas as well as fresh milk from the market). They also reported the total number of microplastics per liter and their color using morphological and chemical characterization such as SEM, EDS, and Raman spectroscopy. Following the protocol above mentioned [27]—also used in the study of Basaran et al. [28]—it was found that the milk samples could not be filtered due to fat content, leading to the filters’ clogging. Enzymatic treatment and alkaline digestion were applied by Buyukunal et al. [29] for the isolation of MPs from Ayran, using enzymatic detergent, ethylenediaminetetraacetic acid (EDTA), and tetramethylammonium hydrate. 

### 3.2. Optical Microscopy

In the current research, the optical microscopy technique revealed the retained MPs on the filter’s surface after the isolation process (Figures S1–S3), and at the same time, they highlighted a variety of colors and shapes that helped to assess the total number of MPs (expressed as microparticles·L^−1^). 

It is important to mention that not only the filters used for milk filtration filters have been the subject of investigations but also the blank ones or those that were used in the reagents’ filtration process in order to ensure that no risk of contamination can occur; the optical microscopy results showed no traces of impurities on their surface at the end of the filtration process; despite the reagents’ risk of contamination, they have also shown no trace of impurities. Table 5 presents the quantification and classification of microparticles according to color (i.e., black, blue, red, brown, gray, yellow, golden, green, and turquoise). Values shown are expressed as microparticles·L^−1^.

After performing optical microscopy on conventional milk samples, the identified microparticles were counted and classified by color. The most microparticles were identified in the L9 sample, and at the opposite pole with the fewest microparticles is the L10 sample (Table 5). The identified colors of microparticles were black (91), blue (20), red (5), brown (4), yellow (2), turquoise (1), and gray (1). Sample L9 presented a variety of microparticles: a total of 36 microparticles of different colors. The black color prevailed and was identified in the ten milk samples analyzed. Blue microparticles were identified in five samples (L1, L3, L5, L8, and L9), while red microparticles were identified in four samples (L2, L5, L9, and L10). Gray, turquoise, and yellow microparticles were undetected in a single sample (L9). The fewest microparticles (2) were identified in sample L10.

The optical microscopy performed on the organic milk samples highlighted four microparticles’ colors (Table 5). The most microparticles were identified in the organic milk sample L7B (99), and the least microparticles were identified in the L11B sample (10). The identified colors of microparticles were black (130), blue (34), red (16), brown (3), and yellow (1). Sample L7B presented a variety of microparticles (99) of different colors. Black and blue-colored microparticles were identified in the six analyzed milk samples. Red and brown microparticles were identified in four samples, while yellow microparticles were identified in one sample (i.e., L7B). In the case of the raw milk category, four samples collected from small producers (LF1, LF2, LF3, and LF4) were analyzed. The most microparticles (Table 5) were identified in sample LF4 (50), and the least microparticles were identified in sample LF1 (7).

The identified colors of microparticles in raw milk samples were black (74), blue (19), red (8), gray (10), yellow (2), brown (1), and green (1). Sample LF3 presented a variety of microparticles of different colors. A green microparticle was identified in the LF2 sample. Black and blue microparticles were identified in all four analyzed milk samples. Red and gray microparticles were identified in three samples, while yellow and brown microparticles were identified only in one sample. 

**Figure 1:**
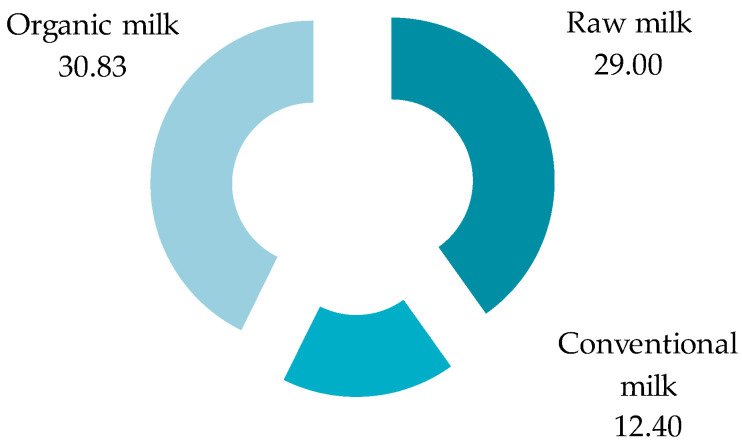
The average number of microparticles (expressed as microparticles·L^−1^) identified in the analyzed milk samples.

Figure 1 shows the results related to the total number of microparticles obtained in the 20 milk samples, which were analyzed in triplicate (i.e., conventional, organic, and raw). In ten conventional milk samples, 124 microparticles (29%), in six organic milk samples, 185 microparticles (44%), and in four raw milk samples, 115 microparticles (27%) were identified. 

In the current research, we achieved the identification of the number, color, and size of the particles, but still, it should be noticed that in the case of the sizes and shapes, the MPs dimensions range from micrometric dots to micro/millimetric twisted fibers. In almost all cases, the MPs showed a compact and opaque state with a high density of matter, while in some cases, they were translucent but still very much compact. After all 48 filters (20 samples, 3 filters/sample, each filter is equivalent to 1 L of milk) had been analyzed and the results were quantified (i.e., round average value), it resulted in a total number of 424 microparticles (Figure 1). In the clean category with average values of <100 microparticles·L^−1^ were Romanian milk with 21.25 microparticles·L^−1^, Mexican milk with 5.9 microparticles·L^−1^ [27], and Ecuadorian milk with 31 microparticles·L^−1^ [30]. In the literature, Da Costa Filho et al. reported the highest value of MPs in milk samples: 2590 microparticles·L^−1^ [31].

### 3.3. Micro-Fourier Transform Infrared Spectroscopy

Vibrational spectroscopy, including Raman spectroscopy and Fourier transform infrared spectroscopy, has become more suitable in MPs analysis, because it provides high-quality polymer identification, even if it is time consuming. Using micro-FTIR imaging, a complete characterization (chemical and morphological, Figures S4–S6) of MPs is achieved, and the obtained results are reliable and reproducible (Figure S7). Differences between the micro-FTIR spectra of MPs isolated from organic/conventional/raw milk samples can be observed relative to a series of spectral regions/peaks highlighted in Figures S8–S10 and Tables S3–S5. After correction for the background spectrum was achieved, all analyzed spectra showed different weak, medium, and strong vibrational frequencies according to the FTIR imaging. In spectroscopic terms, the weak, medium, and strong peaks (intensity), together with wavenumbers, allow the assignment of functional groups from organic compounds [32]. FTIR spectra analysis ranging from 3056 to 3026 cm^−1^ shows the sum of the C-H stretching assigned to the phenyl ring from PE and PS structures, according to data presented in the aforementioned tables; the CH groups of the phenyl ring are strongly associated with strong peaks of 3056 and 3055 cm^−1^ corresponding to polyester, for L5 and L3 conventional milk samples (Table S3), these sample being the only ones that recorded vibrational bands [33,34]. One reason may be due to the cows’ feed, including plastic particles of various origins, and the second reason may be the packaging process. The signals around the region 895 cm^−1^ are assigned to the stretching/bending vibrations of CH, CH_2_, and CH_3_ groups (Tables S3–S5), which are derived from poly(methyl methacrylate), polyamide, polyurethane, and polyester polymers [35,36], some of which have the C-ring. The peaks derived from these vibrational modes are present in almost all analyzed milk samples. The C=O bending of amidic/esteric groups from polyamides/polyester can be assigned peaks around the values 1245–1278 cm^−1^, 1723–1727 cm^−1^, and 1905–1916 cm^−1^ [33,34,36]. Other identified vibrational stretching was the C=C-C aromatic and C-O bonds; the stretching of the C=C-C aromatic bond appears in the region of 1245–1278 cm^−1^, and the C–O bond can be attributed to the peaks from the region of 1206–1055 cm^−1^, corresponding to an esteric group from polyester [34,35]. Weak and medium-intensity bands have been highlighted in all recorded spectra. The region of 1032–891 cm^−1^ is that of the C-H deformation in the plane, and strong intensity bands recorded can be due to C-O stretching. From the analytical point of view, the C-H stretching region (i.e., from 2800 to 3056 cm^−1^) is more meaningful as there are several distinct and assignable absorption differences visible in the spectra (strong and medium peaks, Figures S8–S10). The changes in vibrational frequency can be a result of several different intermolecular and intramolecular interactions which may influence the structure, e.g., the steric effects. Thus, it can be seen from Figures S4–S6 and Tables S3–S5 that several values of the obtained FTIR spectra, expressed as wavenumbers, related to the symmetric CH_2_ stretching mode are rather shifted to higher wavenumbers. In that case, this feature can result in an overlap with the symmetric CH_3_ stretching modes; the overlapping of CH_3_ and CH_2_ therefore can appear as a broadband [33,34,36] according to the spectra presented in Figures S8–S10. The smallest polyester fibers are observed in three conventional milk samples and one organic milk sample in terms of strong peaks around 687–698 cm^−1^ assigned to C-H symmetric stretching from the aromatic ring of polyester.

In all milk samples (i.e., conventional, organic, and raw samples), strong or medium peaks (depending on fat concentration) are observed around 660–667 cm^−1^ due to the stretching vibration of C=O and NH groups mainly assigned to proteins/amino acids from milk (taking into account that samples after isolation were not washed or purified by different processes). In addition, the weak signal of a peak around 1636–1630 cm^−1^ was assigned both to the stretching vibration of the N-H group and the bending vibration of the C-N group from proteins as well, according to the aforementioned hypothesis.

The dominating polymer structures (Tables S3–S5) were assigned to poly(methyl methacrylate, polyamides, polyurethane, polyester, and polyethylene. It is well known that polyethylene and polyamide are common polymer types used for food packages and plastic bottles and can be considered the main source of MPs in foods [34,35]. Other studies show that up to 250 MPs are generated by the opening process of plastic food packages or opening–close processes of plastic bottlenecks/caps [34,35,36]. Other substances transported by MPs identified additives, plasticizers, natural, and non-plastic synthetic fibers, atmospheric deposition, and other inputs (such as proteins from milk).

### 3.4. Scanning Electron Microscopy—Energy-Dispersive X-ray Spectroscopy

**Figure 2:**
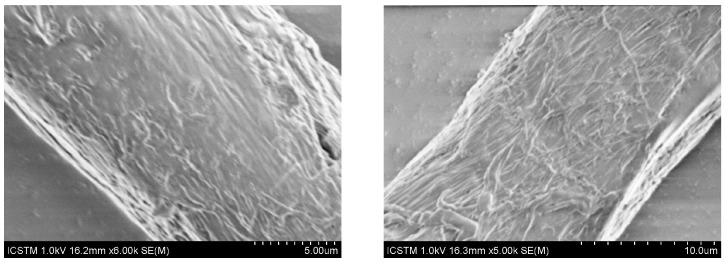
SEM images of the MPs extracted from the analyzed samples.

To perform scanning electron microscopy coupled with energy-dispersive X-ray spectroscopy (SEM-EDS), the MPs were extracted from the surface of the filters and placed on the double-adhesive carbon tape fixed on the support. Figure 2 shows the most representative SEM images obtained for microplastics from conventional and organic milk samples, their morphology being similar. It is characterized by finely textured surfaces, with medium roughness, and presents longitudinal fibers.

**Figure 3:**
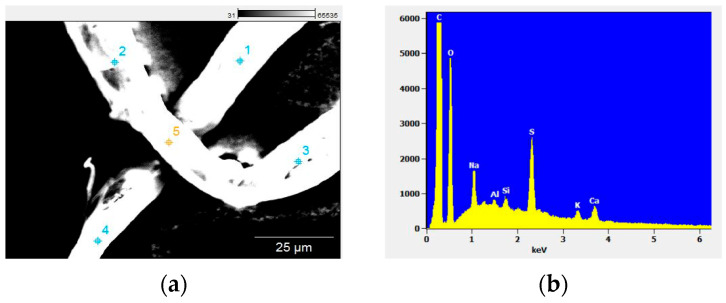
EDS analysis: (**a**) point and shoot technique, 1–5 represent the EDS analysis points; (**b**) EDS spectrum.

The MPs shown in Figure 3a were characterized by an average diameter of 15.5 µm and 2.87 mm in length. The elemental content of MPs was determined using the point and shoot EDS technique (Figure 3b).

The EDS results highlighted the presence of C, O, Na, Mg, Al, Si, P, S, Cl, K, Ca, and Ti. Most of the elements identified in concentrations less than 5% represent milk constituents. The carbon content determined in MPs recorded values in the range of 76.26–97.50% with an average value of 86.18%. The oxygen recorded values were in the range of 0.10–19.70% with an average value of 9.69%, while the amount of sodium was somewhere in the range of 0.23–0.65% with an average value of 0.39%. Values for magnesium ranged from 0.00 to 0.08% with an average value of 0.03%; furthermore, the aluminum showed values in the range of 0.07–0.17% with an average value of 0.12%. Silicon was framed within the range of 0.14–063%, and its average value was 0.28%, while phosphorus reached values between 0.00 and 0.54% with an average value of 0.19%.

Not only magnesium and phosphorus recorded 0.00% but also chlorine and titanium. For these two elements, the highest value reached was 0.08%, respectively, 0.69% and the average value was 0.02% for chlorine and 0.14% for titanium. EDS investigations carried out on MPs showed some interesting values for sulfur as well: the lowest content was 1.03%, while the highest was 3.48%, at an average value of 1.64%. Regarding the calcium content, we can say that the values ranged between 0.48% and 2.94% with an average of 0.29%. The lowest and the highest recorded values for potassium were 0.18%, and 0.55%, respectively, with an average value of 0.29%.

Considering that the purpose of this study was not to identify the source of contamination of milk but its actual contamination, the presence of aluminum and titanium can be explained by the way the milk is packaged, by some pigments used to color the polymer, or by the accidental ingestion of soil by the animals.

### 3.5. High-Performance Liquid Chromatography with Fluorescence Detection

**Figure 4:**
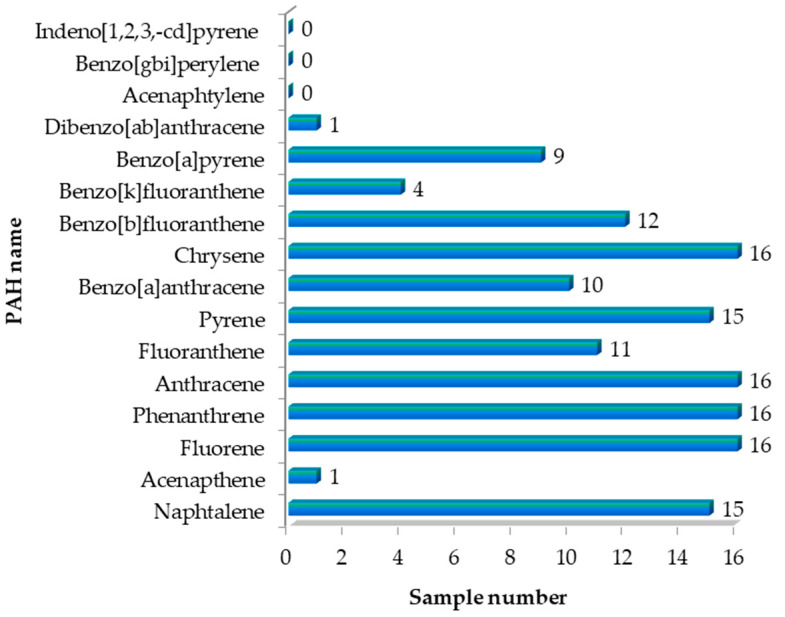
Presentation of the number of samples in which PAHs were identified.

The contamination of milk and dairy products with PAHs is due to both natural and anthropic activities. The high level of PAHs in milk is due to contaminated food or water or due to milk-processing practices [37]. In this respect, from the 16 polycyclic aromatic hydrocarbons provided by the internal standard (Figure S11), 13 PAHs were identified in the 16 conventional and organic milk samples subjected to analysis (Figures S12 and S13); the recorded values are presented in Table S6. Acenaphthylene, benzo[gbi]perylene, and indeno [1,2,3,-cd]pyrene were not identified in the analyzed samples (Figure 4). 

**Figure 5:**
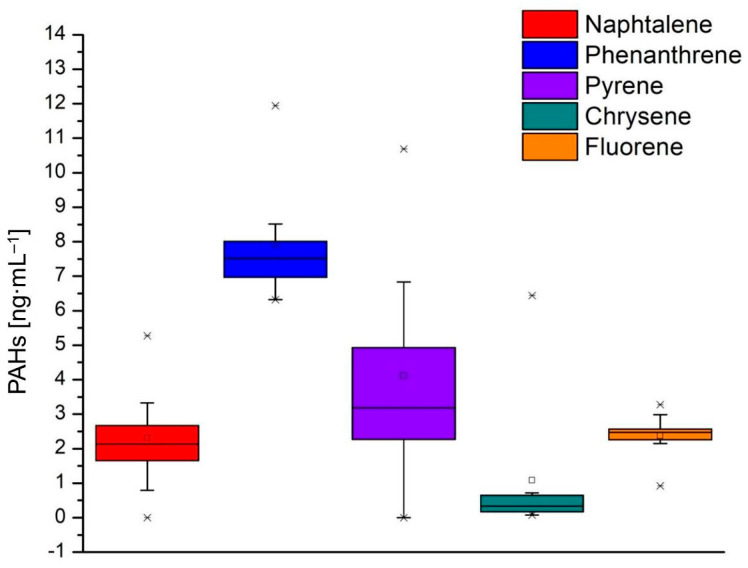
Concentration values of PAHs identified in milk samples: naphthalene, phenanthrene, pyrene, chrysene, and fluorene.

**Figure 6:**
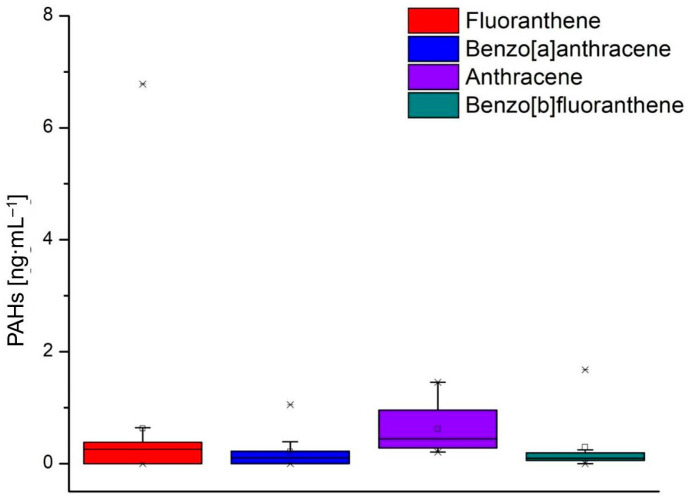
Concentration values of PAHs identified in milk samples: fluoranthene, benzo[a]anthracene, anthracene, and benzo[b]fluoranthene.

**Figure 7:**
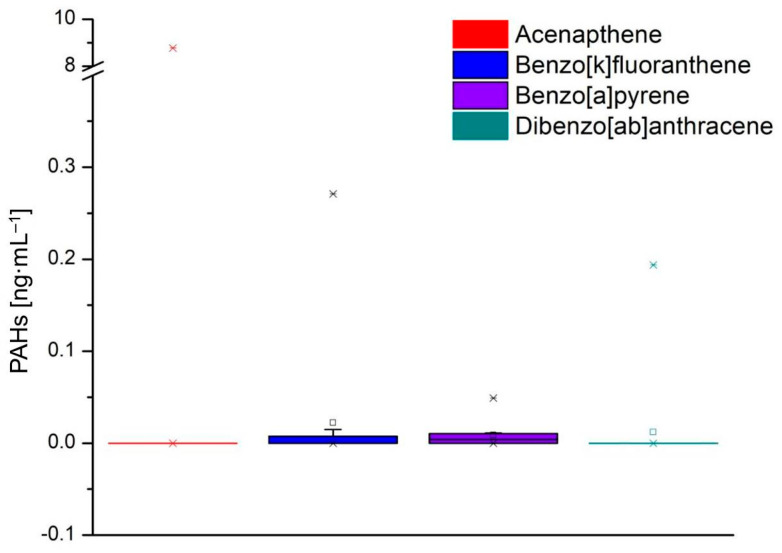
Concentration values of PAHs identified in milk samples: acenaphthene, benzo[k]fluoranthene, benzo[a]pyrene, and dibenzo[a,b]anthracene.

Figure 5, Figure 6 and Figure 7 show the values of the PAHs concentrations identified in the milk samples. The grouping of the compounds in the box-plot graphs was achieved concerning the number of samples in which they were identified and the quantity in which they were found. In addition, in the previously mentioned figures, the minimum, average, and maximum values of the identified concentrations are presented.

According to Figure 5, naphthalene was identified in 15 milk samples, the minimum value identified being 0.797 ng·mL^−1^ (L4) and the maximum value being 5.271 ng·mL^−1^ (L8B). Phenanthrene was identified in 16 milk samples, the minimum value identified being 6.317 ng·mL^−1^ (L7B), and the maximum value being 11.941 ng·mL^−1^ (L6). Pyrene was identified in 15 samples with a minimum value of 2.043 ng·mL^−1^ (L7) and a maximum value of 10.684 ng·mL^−1^ (L5). Chrysene and fluorene were identified in all milk samples analyzed; the minimum value is 0.079 ng·mL^−1^ for chrysene (L8) and 0.925 ng·mL^−1^ (L3) for fluorene, and the maximum value identified was 6.441 ng·mL^−1^ (L6B) for chrysene and 3.278 ng·mL^−1^ (L1B) for fluorene.

Fluoranthene, benzo[a]anthracene, anthracene, and benzo[b]fluoranthrene were identified in 11, 10, 16, and 14 analyzed milk samples, respectively (Figure 6). The minimum value of fluoranthene was 0.081 ng·mL^−1^ (L6B), and the maximum value was 6.779 ng·mL^−1^ (L4). Benzo[a]anthracene values from 10 analyzed milk samples varied between 0.031 ng·mL^−1^ (L1B) and 1.054 ng·mL^−1^ (L6). Anthracene was identified in all analyzed milk samples, but the values were low, falling between 0.206 ng·mL^−1^ (L10B) and 1.455 ng·mL^−1^ (L6). Benzo[b]floranthene was identified in 14 milk samples, the values being between 0.035 ng·mL^−1^ (L10B) and 1.675 ng·mL^−1^ (L6). Figure 7 shows the four PAHs with the lowest values, and one of the four compounds was found in two samples: acenaphthene and dibenzo[ah]anthracene were identified in samples L2 and L8, benzo[k] fluoranthene was identified in four samples (L5, L7, L7B, and L10B) and benzo[a]pyrene was identified in nine samples.

The values recorded for acenaphthene and dibenzo[a,b]anthracene were 8.772 ng·mL^−1^ and 0.194 ng·mL^−1^. The content of benzo[k]fluoranthene in the samples varied between 0.015 ng·mL^−1^ (L2) and 0.271 ng·mL^−1^ (L4). For benzo[a]pyrene, the minimum value recorded was 0.004 ng·mL^−1^ for samples L5, L10, and L6B, and the maximum value was recorded in sample L5.

### 3.6. Inductively Coupled Plasma Mass Spectrometry

The first step in ICP–MS analysis of heavy metals (i.e., Cr, Cd, Pb, Ni, Cu, Mn, Zn, and Sr) was the assessment of the analytical sensitivity of the method by calculating the limit of detection (LOD) and limit of quantitation (LOQ). LOD and LOQ are the terms used to describe the smallest content that can be reliably detected or measured by an analytical procedure [38]. For this part of the study, seven blank solutions were prepared and analyzed by the same protocols as the samples. The values obtained for HMs content in blank solutions were used in Equations (4) and (5) to calculate LOD and LOQ:

(4) $$LOD=\bar{C_{blank}}+3\cdot SD$$

(5) $$LOQ=\bar{C_{blank}}+10\cdot SD$$

where $\bar{C_{blank}}$ represents the average value of blank content and SD is the standard deviation calculated for these values.

The values calculated for LOD and LOQ were in the ranges of 2.242–10.954 μg·L^−1^ and 4.441–28.295 μg·L^−1^, respectively (the results are presented in Table 6).

Taking into account the fact that ICP-MS is a semi-quantitative method, it was necessary to determine the method accuracy using the standard reference material, SRM NIST 1869—Infant/Adult Nutritional Formula II. The ratio between the recovered value and certified value (expressed as percentage, %) represents the recovery percentage of the method. The acceptable values for recovery ranged between 80 and 110% for an analyte content of 1 ppm according to Hubber [39]. Therefore, the developed ICP-MS method was accurate for the quantitation of Cr, Mn, Cu, and Zn in milk—the obtained values for recovery percentage are shown in Table 7.

(6) Recovery [%]=(Recovered value)/(Certified value)·100

**Figure 8:**
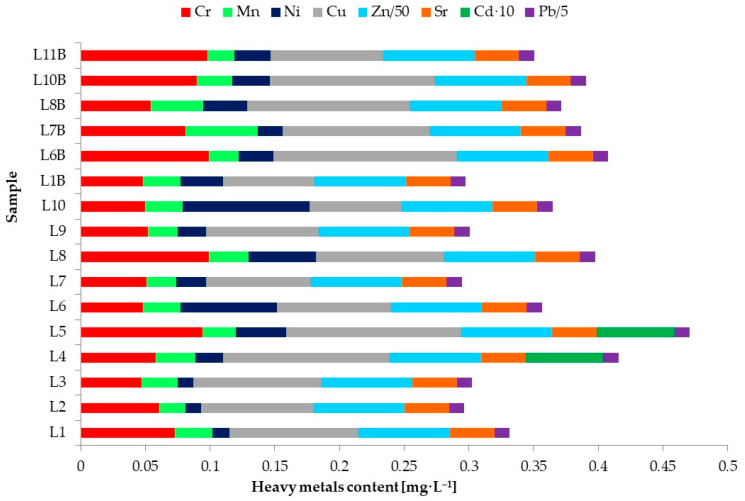
Heavy metal content in milk samples determined by ICP–MS.

Table 8 shows the maximum limits allowed for the heavy metals determined in the samples subjected to ICP-MS analysis. The content of metals in conventional and organic milk samples determined by ICP–MS is shown in Figure 8. Chromium, manganese, nickel, copper, zinc, strontium, cadmium, and zinc were determined in all 16 milk samples subjected to analysis (Table S7). Cadmium was identified in two samples according to data presented in Table S7.

The contents of heavy metals in some milk samples were quantified using the developed ICP-MS method. The levels of Cr, Mn, Ni, Cu, Zn, Sr, Cd, and Pb in conventional milk samples were found to be in the ranges of 0.047–0.099 mg·L^−1^, 0.020–0.031 mg·L^−1^, 0012–0.098 mg·L^−1^, 0.071–0.135 mg·L^−1^, 3.069–4.082 mg·L^−1^, 0.306–0.615 mg·L^−1^, <LOD–0.011 mg·L^−1^, and 0.016–0.466 mg·L^−1^, respectively. For the organic milk samples, the levels of Cr, Mn, Ni, Cu, Zn, Sr, and Pb were found to be in the ranges of 0.048–0.099 mg·L^−1^, 0.021–0.056 mg·L^−1^, 0019–0.034 mg·L^−1^, 0.071–0.142 mg·L^−1^, 3.268–3.803 mg·L^−1^, 0.207–0.531 mg·L^−1^, and 0.042–0.071 mg·L^−1^, respectively. The values for the Cd content were under the LOD value. The maximum values of HMs were recorded as follows: Cr (L8-conventional and L6B-organic), Mn (L4-conventional and L7B-organic), Ni (L10-conventional and L8B-organic), Cu (L5-conventional and L6B-organic), Zn (L5-conventional and L8B-organic), Sr (L10-conventional and L10B-organic), Cd (L5-conventional), and Pb (L6-conventional and L8B-organic) (Figure 8 and Table S7).

The Codex Alimentarius and EC Regulation 1881/2006 regulates the maximum level of lead in milk at 0.020 mg·L^−1^ (Table 8); the other metals (i.e., As, Hg) provided in the two reference documents have not been determined. Compared with these international standards, all analyzed samples (except the L3 sample) recorded lead values higher than the maximum level for both conventional and organic samples. In terms of national legislation, Order 975/1998 establishes and approves the sanitary hygiene norms for food, including maximum levels of Cu, Zn, Cs, and Pb in milk. Compared with this national order, the analyzed samples recorded values under the maximum thresholds for Cu and Zn, while they exceeded the thresholds of Cd (L5-conventional) and Pb (L6, L7, L8, L9, and L10—all conventional milk samples).

### 3.7. Data Analysis

Exposure to microplastics poses a serious health risk to humans, particularly for children (0–3 years of age). This is because the chemicals that are added to plastic are interfering with hormones at such young ages, which leads to disease. MPs pass through the digestive system without any issues and end up in the feces; they can be found in various body organs, penetrate cell membranes and end up in the blood, where they are then passed from pregnant mothers to developing fetuses through the placenta. More information about MPs can be found in the Data Analysis section of the Supplementary Materials.

The water and feed for the animals is undoubtedly one of the contamination sources. This process may have an implicit impact on the milk’s quality and provide an explanation for the presence of microplastics in it. The wrapping used in the milk processing facilities could be another factor. In light of this, the results on health risk consumption linked to MPs and HMs can help gain a better grasp of the seriousness of the global phenomenon known as pollution, which has detrimental effects on both adult and pediatric health.

The concentration of microplastics in the milk samples was used to estimate the pollution load index and the estimated daily intake, while the heavy metals content was used to assess the estimated daily intake and health risk index (for both adults and children). The obtained results are presented in the following subsections.

#### 3.7.1. Pollution Load Index (PLI) of Microplastics

**Figure 9:**
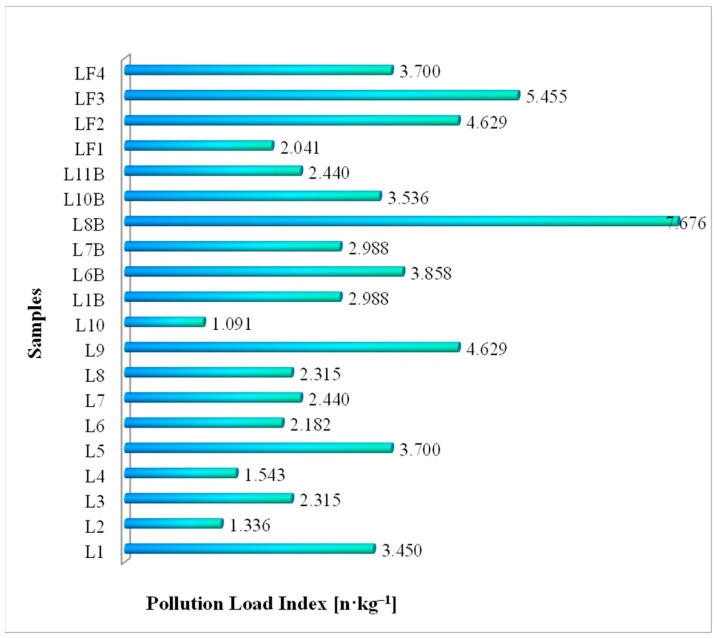
Pollution load index (PLI) for conventional, organic, and raw analyzed milk samples.

The loading index of microplastic pollution of the 20 milk samples was calculated based on Table 5 and according to Equation (1). The obtained results are presented in Figure 9.

For the microplastic pollution load index for conventional milk samples, the minimum calculated value was 1.091 n·kg^−1^ (L10), while the maximum value was 4.629 n·kg^−1^ (L9); for organic milk samples, the minimum calculated value was 2.440 n·kg^−1^ (L11B), while the maximum value determined was 7.676 n·kg^−1^ (L8B). In addition, in raw milk, the minimum value was 2.041 n·kg^−1^ (LF1), while the maximum value was 5.455 n·kg^−1^ (LF3). Concerning the risk level criteria for the microplastic pollution load index (Table 4), the 20 analyzed milk samples fall into the very low-risk category. 

#### 3.7.2. Estimated Daily Intake of MPs

**Figure 10:**
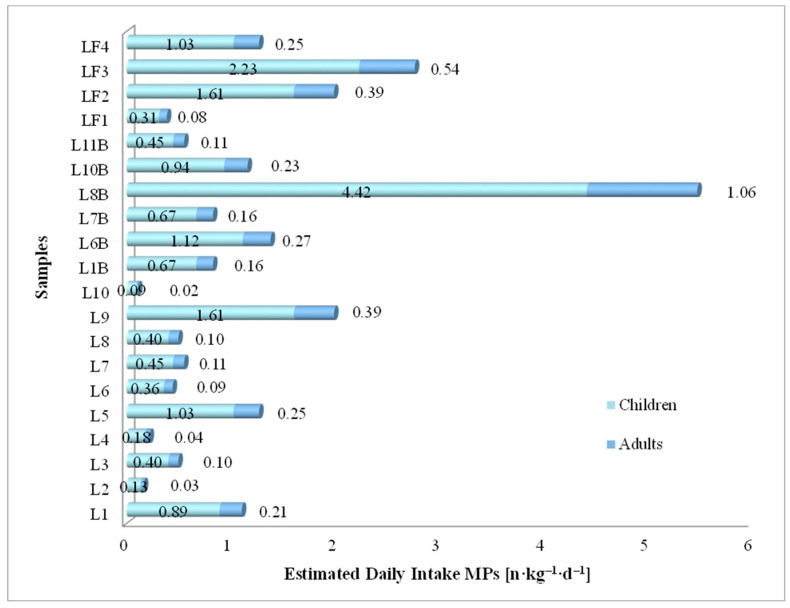
Estimated daily intake MPs values for adults and children from milk samples.

The estimated daily intake of MPs for adults and children (Figure 10) during milk consumption (conventional, organic, and raw) was calculated using Equation (2). The recommended daily dose for adults is 750 mL of milk, and for children, it is 650 mL of milk. 

The estimated daily intake of MPs from conventional milk recorded values between 0.09 and 1.61 n·kg^−1^·d^−1^ for children in samples L10 and L9, and for adults, the values were recorded between 0.02 and 0.39 n·kg^−1^·d^−1^ for samples L10 and L9. For organic milk, the EDI values for children ranged between 0.45 and 4.42 n·kg^−1^·d^−1^ for samples L11B and L8B, and for adults, the values ranged between 0.11 and 1.06 n·kg^−1^·d^−1^ in samples L11B and L8B. In the case of the four raw milk samples, the EDI values for children varied from 0.31 to 2.23 n·kg^−1^·d^−1^ in samples LF1 and LF3, while for adults, the EDI values ranged from 0.08 to 0.54 n·kg^−1^·d^−1^ for the same samples. Milk samples L10, L11B, and LF1 recorded the lowest values for EDI due to the small number of MPs (Table 5). The L8B sample recorded the highest values among the 20 milk samples analyzed in triplicate, while at the opposite pole was the L10 sample. 

#### 3.7.3. Estimated Daily Intake of Metals 

Based on Equation (3), which establishes the estimated daily intake of heavy metals from conventional and organic milk, the obtained results are presented in Table 9; the minimum and maximum values determined for each metal are assigned for both children and adult consumption. The estimated daily intake of heavy metals represents the ratio of the estimated daily consumption to the body weight, which for children aged 10 years is 14 kg and for adults aged 70 years is 70 kg. The minimum value for the amount of chromium that could be ingested by children with a milk consumption of 625 mL·day^−1^ is 0.00211 mg·kg^−1^·day, and the maximum value is 1.50000 mg·kg^−1^·day. In adults for a milk consumption of 750 mL·day^−1^, the minimum value of chromium ingested is 0.00051 mg·kg^−1^·day, and the maximum value is 0.00107 mg·kg^−1^·day. For manganese, the estimated daily intake for children ranges from 0.00091 to 0.14000 mg·kg^−1^·day, and for adults, it ranges from 0.00022 to 0.00060 mg·kg^−1^·day. For nickel, the minimum value that could be ingested is 0.00052 mg·kg^−1^·day, and the maximum value is 0.02000 mg·kg^−1^·day, and for adults, the minimum ingested value is 0.00012 mg·kg^−1^·day, and the maximum value is 0.00105 mg·kg^−1^·day. For copper, the estimated daily intake for children is in the range of 0.00315–0.00632 mg·kg^−1^·day, and for adults, it is in the range of 0.00076–0.00152 mg·kg^−1^·day. For zinc, the estimated daily intake for children is in the range of 0.13703–0.30000 mg·kg^−1^·day, and for adults, it is in the range of 0.03289–0.04374 mg·kg^−1^·day.

For lead, the daily amount ingested by children is in the range of 0.00070–0.01391 mg·kg^−1^·day, and for adults, the lead intake is in the range of 0.00017–0.00500 mg·kg^−1^·day. The daily consumption of cadmium was determined only in two samples; for the others, the values were below the detection limit.

#### 3.7.4. Health Risk Index

The health risk index was calculated based on the estimated daily intake of heavy metals and the reference oral dose, and the recorded results are presented in Table 10. Heavy metals, including Cr, Cd, Pb, Mn, Ni, Cu, and Zn, were determined in all milk samples, but the obtained values did not exceed the maximum allowed limit (MAL 1.00).

#### 3.7.5. Hierarchical Cluster Analysis

**Figure 11:**
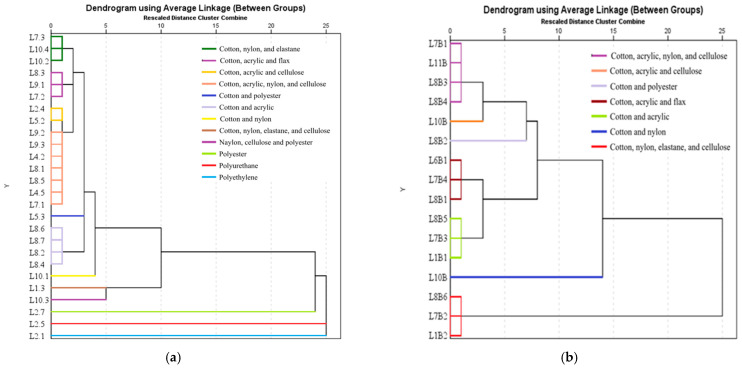

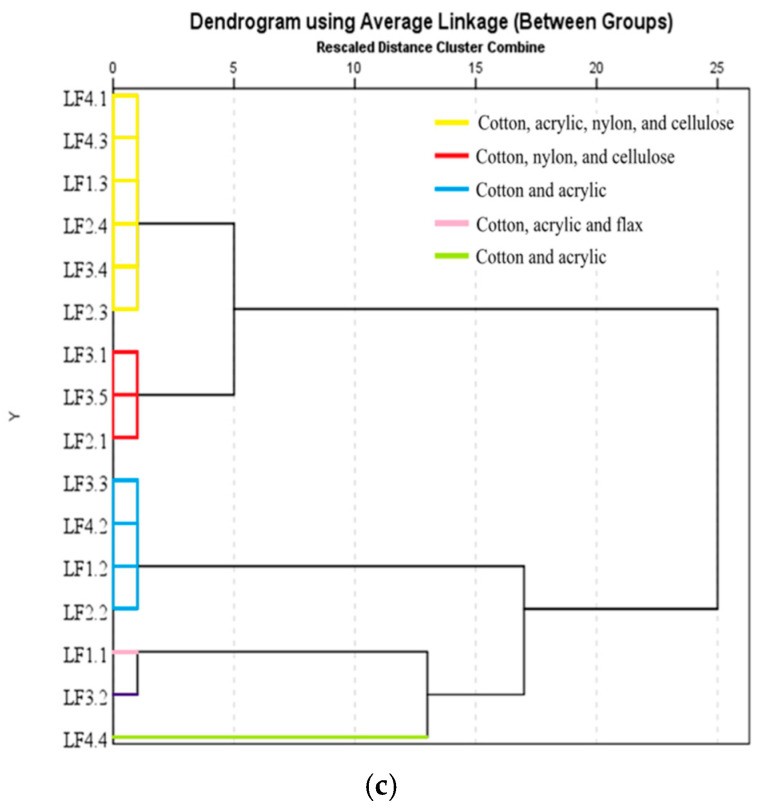
Dendrogram plot tab—distribution of MPs from (**a**) conventional, (**b**) organic, and (**c**) raw milk samples.

One method used to determine the relatively homogeneous groups based on predefined characteristics (MPs composition) is hierarchical cluster analysis. The hierarchical groups created for the milk categories (conventional, organic, and raw) are shown in Figure 11, with the type and nature of microparticles serving as default variables. The dendrograms for each milk category were made up of microplastics as follows: 27 MPs from conventional milk and 16 MPs from organic and raw milk. In the case of conventional milk, the cluster analysis grouped the 25 MPs in conjunction with Table S3 into ten distinct clusters, while the 16 MPs from organic and raw milk in conjunction with Tables S4 and S5 were grouped into 7 and 5 clusters, respectively. MPs from the three categories of milk have in their composition polymeric compounds such as poly(methyl methacrylate, polyamides, polyurethane, polyester, polyethylene, as well as natural organic compounds such as cotton, cellulose, and flax.

It can be concluded that the potential sources of the appearance of MPs in milk can be the textile materials used in the process of sanitizing and cleaning the udder of animals, the clothing of the workers who operate and handle the raw material (milk) from the farms where the milk comes from, throwing waste in undeveloped spaces (ponds, rivers, plains), and plastic packaging used for packaging finished products. The presence of MPs in food products is not only caused by the technological process of obtaining food or from packaging following the exfoliation of the plastic material that protects the product. Currently, milk factories and dairy products tend to automate the technological process so that the operators (employees) come into contact with the food products as little as possible throughout the technological process. Food industry equipment is made of food safety-certified stainless steel. Work equipment (including capes/caps, protective helmets, or ear protection devices in places where the permissible decibel limit is exceeded), according to the standards applied in the food industry and operator hygiene, is carried out before entering or leaving the work sections. On the other hand, several studies [40,41,42,43,44,45] revealed that microplastics act as a carrier for other emerging contaminants such as PAHs and HMs, posing a higher and uncontrolled risk to ecosystem and human health. In a recent study, Ali et al. [40] revealed that the mechanism of co-occurrence and the role of MPs as carriers in the distribution of PAHs in the marine systems and then in the trophic chain is essential to understand the risk of exposure to these contaminants. Other authors, Lee and Jeoung [41], in their study tried to answer the question “How dangerous microplastics are to the human body”. But the most correct answer was given by Leslie et al. [42] two years earlier, who quantified the presence of extremely small pieces, invisible to the naked eye, in human blood, drawing the world’s attention for the first time to the possibly carcinogenic effects of MPs as well as the possibility that these microparticles play an important role as carriers for other emerging contaminants. Moreover, Raguza et al. [43] highlighted for the first time in the world the presence of MPs in the human placenta. The research was supported two years later by Zhu et al. [44]. Part of the explanations regarding the presence of these MPs carriers in human blood, in the human placenta, were explained by Raguza et al. [45] through their studies that highlighted the presence of MPs in breast milk. All these studies fully support the assumption of our research regarding the presence of MPs in milk and the affinity they can have with other emerging contaminants, such as PAHs and HMs, through strong physicochemical interactions. The carriers behavior of MPs for PAHs and HMs is influenced by several factors related to MPs, PAHs, and HMs characteristics (i.e., type, size, shape, roughness and chemical composition) and those related to the environment (i.e., pH, salinity, acidity, temperature, UV light, ionic strength, matrix type, etc.).

## 4. Conclusions

The appearance of emerging contaminants such as PAHs and MPs in the environment implicitly creates a risk to biodiversity and life mainly because in the natural environment, organic compounds can degrade into new toxic organic intermediaries (in the case of PAHs) or can decompose into extremely small fragments, sometimes invisible, through various mechanical procedures, UV radiation or thermal oxidation, etc. (in case of MPs). In addition to irregular fragmented particles, synthetic fibers constitute a significant share of microplastics, often exceeding other types of particles such as fragments and beads, the consequence being clearly determined by domestic activity, industrial activity, fishing activity, food industry, etc. Unlike irregular or spherical particles, fibers are widely neglected in current research, but without a doubt, they represent a risk to humans, especially to children. The study of MPs particle sizes, in conjunction with analytical investigations regarding the chemical composition of MPs, as well as with the risk effect of compositionally modified dairy products by identifying microplastic particles of small/different sizes on human health, is an assumed objective of the present research. In addition, the research related to the methods of food preparation, breeding, processing, transport, geographical conditions, etc., are analyzed statistically and will contribute in the future to the establishment of well-founded hypotheses and interpretations regarding the source–cause–effect relationship of MPs. The origin of the microplastics found in milk was not the subject of this study, but taking into account the fact that they were detected both in organic and conventional samples, as well as in raw milk samples, we can say that the basis of the contamination can mainly be the feed and drinking water, but we do not exclude contamination during processing and the materials used in the packaging. The present study wanted to establish a unique protocol for microplastics isolated from milk regardless of its nature, and it remains for future studies to include the cause of the contamination as a starting point. 

A total of 425 MPs were recovered from the 20 analyzed milk samples. MPs had varied sizes (68–2152 μm—Tables S3–S5) and colors (black, blue, red, brown, gray, yellow, golden, green, and turquoise—Table 5), and five polymers were identified (i.e., poly(methyl methacrylate), polyamides, polyurethane, polyester, and polyethylene—Tables S3–S5). The profile of the MPs isolated from milk microplastics was established by optical microscopy and micro-FTIR as fiber (74.14%), fragment/irregular (22.37%), and oval (3.49%)—according to data presented in Tables S3–S5. The MP concentrations—expressed as mean value ± standard deviation—varied between 12.40 ± 10.76 MPs·L^−1^ (in conventional milk samples) and 30.86 ± 33.80 MPs·L^−1^ (in organic milk samples).

The microplastic pollution load index values recorded in conventional milk samples were between 1.091 and 4.629 n·L^−1^; in organic milk samples, it ranged between 2.440 and 7.676 n·L^−1^; in raw milk samples, it ranged between 2.041 and 5.455 n·L^−1^ (Figure 9). The human risk of MPs intake through the consumption of milk expressed as estimated daily intake ranged from 0.09 to 4.42 n·kg^−1^·d^−1^ for children, while for adults, the values ranged from 0.02 to 1.06 n·kg^−1^·d^−1^ (Table 9, Figure 10).

The values of the health risk index induced by heavy metals (Cr, Cd, Pb, Mn, Ni, Cu, and Zn) ingested from milk did not exceed the maximum allowed limit (i.e., 1.00)—see Table 10. Although all analyzed samples do not represent a risk to the health of children and adults, this complex food must be continuously monitored, and the values must be presented on the packaging for the correct consumer information.

This research brings added value on one hand due to the highly innovative isolation method of MPs from milk and dairy products, and on the other hand due to the complex investigation that for the first time brings together three contaminants of emerging concerns (i.e., MPs, PAHs, and HMs), which were first investigated separately at the beginning and then together in terms of humans health risk for both children and adult categories. 

## 5. Patents

Patent applications no. PCT/RO2024/000010/15.04.2024 and RO137927A0/26.09.2023—Rapid Method for isolation of microplastics from milk, yogurt, sour cream, and butter, authors Radulescu, C.; Dulama, I.D.; Banica, A.L.; Bucurica, I.A.; Stirbescu, R.M.; Gorghiu, L.M. 

## Figures and Tables

**Table 1:** Digestion steps and parameters.

Parameters/Step	1	2	3
Temperature [°C]	145	170	190
Pressure [bar]	40	40	40
Power [%]	80	80	80
Ramp [min]	2	5	2
Time [min]	5	10	15

**Table 2:** The risk level criteria for the pollution load index of microplastics [19].

PLI	Hazard Level
<10	Very low hazard
10–20	Low hazard
20–30	Medium hazard
>30	High hazard
-	Very high hazard

**Table 3:** The values of parameters for the *EDI* calculation.

	Ir [kg·d^−1^]	Ef [d·y^−1^]	Ed [y]	Bw [kg]	At [d]
Adults	0.750	365	70	70	25,550
Children	0.625	365	10	14	3650

**Table 4:** Oral reference dose for the analyzed metals (expressed as mg·kg^−1^·day) [22,23,24].

	Cr	Mn	Ni	Cu	Zn	Sr	Cd	Pb
*R_f_D*	1.500	0.140	0.020	0.040	0.300	0.600	0.001	0.035

**Table 5:** Optical microscopy on milk samples.

Sample Code	Color and Number of Microparticles									Total [Microparticles·L^−1^]
	Black	Blue	Red	Brown	Gray	Yellow	Golden	Green	Turquoise	
Conventional milk										
L1	12	8	nd *	nd *	nd *	nd *	nd *	nd *	nd *	20
L2	2	nd *	1	nd *	nd *	nd *	nd *	nd *	nd *	3
L3	8	1	nd *	nd *	nd *	nd *	nd *	nd *	nd *	9
L4	4	nd *	nd *	nd *	nd *	nd *	nd *	nd *	nd *	4
L5	15	3	1	4	nd *	nd *	nd *	nd *	nd *	23
L6	8	nd *	nd *	nd *	nd *	nd *	nd *	nd *	nd *	8
L7	10	nd *	nd *	nd *	nd *	nd *	nd *	nd *	nd *	10
L8	7	2	nd *	nd *	nd *	nd *	nd *	nd *	nd *	9
L9	24	6	2	nd *	1	nd *	2	nd *	1	36
L10	1	nd *	1	nd *	nd *	nd *	nd *	nd *	nd *	2
Organic milk										
L1B	7	6	2	nd *	nd *	nd*	nd *	nd *	nd *	15
L6B	18	4	2	1	nd *	nd*	nd *	nd *	nd *	25
L7B	9	3	2	1	nd *	nd*	nd *	nd *	nd *	15
L8B	71	16	10	1	nd *	1	nd *	nd *	nd *	99
L10B	18	2	nd *	1	nd *	nd *	nd *	nd *	nd *	21
L11B	7	3	nd *	nd *	nd *	nd *	nd *	nd *	nd *	10
Raw milk										
LF1	2	3	nd *	nd *	2	nd *	nd *	nd *	nd *	7
LF2	21	5	4	nd *	5	nd *	nd *	1	nd *	36
LF3	33	8	2	1	4	2	nd *	nd *	nd *	50
LF4	18	3	2	nd *	nd *	nd *	nd *	nd *	nd *	23

nd *—unidentified.

**Table 6:** Calculated LOD and LOQ values for seven blank solutions (expressed in μg·L^−1^) to verify the sensitivity of the ICP-MS method.

	Cr	Mn	Ni	Cu	Zn	Sr	Cd	Pb
LOD	2.766	5.337	4.041	11.073	9.741	2.488	2.242	10.954
LOQ	6.556	14.871	10.284	24.926	27.449	5.824	4.441	28.295

**Table 7:** Recovery of ICP-MS method by using SRM NIST 1869–Infant/Adult Nutritional Formula II.

Metals	Certified Value [mg/kg]	Determined Value [mg/kg]	Recovery [%]
Cr	0.859 ± 0.066	0.861 ± 0.001	100.23
Mn	46.000 ± 1.600	44.577 ± 2.061	96.91
Cu	19.000 ± 0.380	18.937 ± 0.261	99.67
Zn	144.000 ± 3.200	144.226 ± 3.745	100.16

**Table 8:** The maximum allowed limit for certain contaminants in food products.

Code	Cr	Mn	Ni	Cu	Zn	Sr	Cd	Pb
MAL ^1^	nr *	nr *	nr *	nr *	nr *	nr *	nr *	0.020
MAL ^2^	nr *	nr *	nr *	0.500	5.000	nr *	0.010	0.100

nr *—regulated values; MAL ^1^—Codex Alimentarius General Standard for Contaminants and Toxins in Food and Feed (STAN 193-1995) and EC Regulation 1881/2006 establishing maximum levels for certain contaminants in food products; MAL ^2^—Order 975/1998 on the approval of hygienic–sanitary norms for food.

**Table 9:** The estimated daily intake (EDI) of heavy metals from conventional and organic milk samples related to both children and adults categories.

EDI	Sample Cod	Cr	Mn	Ni	Cu	Zn	Sr	Cd	Pb
Children	L1	0.00324	0.00130	0.00060	0.00447	0.15703	0.01539	nd	0.00263
	L2	0.00274	0.00091	0.00052	0.00386	0.13703	0.01410	nd	0.00218
	L3	0.00211	0.00125	0.00052	0.00443	0.15129	0.01662	nd	0.00070
	L4	0.00258	0.00138	0.00095	0.00576	0.15964	0.01802	0.00025	0.00376
	L5	0.00417	0.00114	0.00175	0.00603	0.18223	0.02067	0.00047	0.00386
	L6	0.00216	0.00131	0.00334	0.00393	0.16221	0.01884	nd	0.02082
	L7	0.00229	0.00104	0.00103	0.00361	0.16636	0.01896	nd	0.00838
	L8	0.00441	0.00140	0.00233	0.00441	0.16749	0.01955	nd	0.00891
	L9	0.00232	0.00105	0.00098	0.00390	0.17325	0.01368	nd	0.00733
	L10	0.00222	0.00130	0.00437	0.00315	0.16507	0.02747	nd	0.01391
	L1B	0.00212	0.00129	0.00148	0.00319	0.14590	0.00923	nd	0.00233
	L6B	0.00444	0.00101	0.00121	0.00632	0.15935	0.02077	nd	0.00228
	L7B	0.00360	0.00250	0.00083	0.00511	0.16469	0.02205	nd	0.00218
	L8B	0.00242	0.00183	0.00151	0.00561	0.16978	0.01693	nd	0.00319
	L10B	0.00432	0.00119	0.00130	0.00574	0.15613	0.02372	nd	0.00287
	L11B	0.00439	0.00096	0.00127	0.00390	0.16617	0.01472	nd	0.00185
Adults	L1	0.00078	0.00031	0.00014	0.00107	0.03769	0.00369	nd	0.00063
	L2	0.00066	0.00022	0.00012	0.00093	0.03289	0.00339	nd	0.00052
	L3	0.00051	0.00030	0.00012	0.00106	0.03631	0.00399	nd	0.00017
	L4	0.00062	0.00033	0.00023	0.00138	0.03831	0.00432	0.00006	0.00090
	L5	0.00100	0.00027	0.00042	0.00145	0.04374	0.00496	0.00011	0.00093
	L6	0.00052	0.00031	0.00080	0.00094	0.03893	0.00452	nd	0.00500
	L7	0.00055	0.00025	0.00025	0.00087	0.03993	0.00455	nd	0.00201
	L8	0.00106	0.00034	0.00056	0.00106	0.04020	0.00469	nd	0.00214
	L9	0.00056	0.00025	0.00023	0.00093	0.04158	0.00328	nd	0.00176
	L10	0.00053	0.00031	0.00105	0.00076	0.03962	0.00659	nd	0.00334
	L1B	0.00051	0.00031	0.00035	0.00077	0.03502	0.00222	nd	0.00056
	L6B	0.00107	0.00024	0.00029	0.00152	0.03824	0.00498	nd	0.00055
	L7B	0.00086	0.00060	0.00020	0.00123	0.03952	0.00529	nd	0.00052
	L8B	0.00058	0.00044	0.00036	0.00135	0.04075	0.00406	nd	0.00077
	L10B	0.00104	0.00029	0.00031	0.00138	0.03747	0.00569	nd	0.00069
	L11B	0.00105	0.00023	0.00030	0.00094	0.03988	0.00353	nd	0.00044

nd—unidentified.

**Table 10:** Calculated values of health risk index (HRI) for heavy metals in milk samples related to both children and adults categories.

HRI	Sample Cod	Cr	Mn	Ni	Cu	Zn	Sr	Cd	Pb
Children	L1	0.002	0.009	0.030	0.112	0.523	0.026	nd	0.075
	L2	0.002	0.007	0.026	0.097	0.457	0.024	nd	0.062
	L3	0.001	0.009	0.026	0.111	0.504	0.028	nd	0.020
	L4	0.002	0.010	0.047	0.144	0.532	0.030	0.253	0.107
	L5	0.003	0.008	0.087	0.151	0.607	0.034	0.471	0.110
	L6	0.001	0.009	0.167	0.098	0.541	0.031	nd	0.595
	L7	0.002	0.007	0.051	0.090	0.555	0.032	nd	0.239
	L8	0.003	0.010	0.116	0.110	0.558	0.033	nd	0.254
	L9	0.002	0.007	0.049	0.097	0.577	0.023	nd	0.209
	L10	0.001	0.009	0.218	0.079	0.550	0.046	nd	0.397
	L1B	0.001	0.009	0.074	0.080	0.486	0.015	nd	0.067
	L6B	0.003	0.007	0.060	0.158	0.531	0.035	nd	0.065
	L7B	0.002	0.018	0.041	0.128	0.549	0.037	nd	0.062
	L8B	0.002	0.013	0.076	0.140	0.566	0.028	nd	0.091
	L10B	0.003	0.009	0.065	0.143	0.520	0.040	nd	0.082
	L11B	0.003	0.007	0.064	0.097	0.554	0.025	nd	0.053
Adults	L1	0.001	0.002	0.007	0.027	0.126	0.006	nd	0.018
	L2	0.000	0.002	0.006	0.023	0.110	0.006	nd	0.015
	L3	0.000	0.002	0.006	0.027	0.121	0.007	nd	0.005
	L4	0.000	0.002	0.011	0.035	0.128	0.007	0.061	0.026
	L5	0.001	0.002	0.021	0.036	0.146	0.008	0.113	0.026
	L6	0.000	0.002	0.040	0.024	0.130	0.008	nd	0.143
	L7	0.000	0.002	0.012	0.022	0.133	0.008	nd	0.057
	L8	0.001	0.002	0.028	0.026	0.134	0.008	nd	0.061
	L9	0.000	0.002	0.012	0.023	0.139	0.005	nd	0.050
	L10	0.000	0.002	0.052	0.019	0.132	0.011	nd	0.095
	L1B	0.000	0.002	0.018	0.019	0.117	0.004	nd	0.016
	L6B	0.001	0.002	0.015	0.038	0.127	0.008	nd	0.016
	L7B	0.001	0.004	0.010	0.031	0.132	0.009	nd	0.015
	L8B	0.000	0.003	0.018	0.034	0.136	0.007	nd	0.022
	L10B	0.001	0.002	0.016	0.034	0.125	0.009	nd	0.020
	L11B	0.001	0.002	0.015	0.023	0.133	0.006	nd	0.013

nd—unidentified.

## Data Availability

The original contributions presented in the study are included in the article and Supplementary Material, further inquiries can be directed to the corresponding author.

## Supplementary Materials

The following supporting information can be downloaded at https://www.mdpi.com/article/10.3390/foods13193069/s1. Figure S1. Optical microscopy images of representative MPs identified in conventional milk; Figure S2. Optical microscopy images of representative MPs identified in organic milk; Figure S3. Optical microscopy images of representative MPs identified in raw milk; Figure S4. Measurement sequence of microparticles identified in conventional milk samples; Figure S5. Measurement sequence of microparticles identified in organic milk samples; Figure S6. Measurement sequence of microparticles identified in raw milk samples; Figure S7. Natural and synthetic MPs from the analyzed milk samples; Figure S8. FTIR spectra of MPs from conventional milk samples (selection): (a) polyethylene; (b) polyester; (c) polyurethane; Figure S9. FTIR spectra of MPs from organic milk samples (selection): (a) mixture: cotton, nylon, and cellulose; (b) mixture: cotton and acrylic; Figure S10. FTIR spectra of MPs from raw milk samples (selection): (a) mixture: cotton, acrylic, and flax; (b) mixture: cotton, cotton, cellulose, nylon, and acrylic; (c) cotton and acrylic; Figure S11. Calibration curve related to 16 PAHs using HPLC-FLD; Figure S12. Chromatograms of PAHs in conventional milk samples; Figure S13. Chromatograms of PAHs in organic milk samples; Table S1. General presentation of conventional milk samples; Table S2. General presentation of organic milk samples; Table S3. Identification of MPs according to the spectra library of OPUS software v.7.5 (conventional milk samples); Table S4. Identification of MPs according to the spectra library of OPUS software v.7.5 (organic milk samples); Table S5. Identification of MPs according to the spectra library of OPUS software v.7.5 (raw milk samples); Table S6. The concentration of PAHs in conventional and organic milk samples; Table S7. The content of heavy metals in milk samples determined by ICP-MS [mg·L^–1^ ± SD].

## Appendix A

**Table A1:** The parameters tested for the homogenization process and the observed effects.

	Time [min]	10	30	60
Speed [rpm]				
50		VFG	VFG	VFG
100		AH	AH	GH
150		GH	GH	GH

VFG = visible fat globules/low homogeneity; AH = average homogeneity; GH = good homogeneity.

## Appendix B

**Table A2:** The parameters tested for the digestion process and the observed effects.

	Time [min]	20	30	60	90
Temperature [°C]					
~21		LD	LD	MD	MD
30		GD	GD	GD	GD
45		GD	GD	GD	GD
60		GD	GD	GD	GD
75		GD	GD	GD	GD

LD = low digestion/samples cannot be filtered; MD = medium digestion/samples are not completely filtered; GD = good digestion/samples are completely filtered.

## References

[1] Pathak H.K., Seth C.S., Chauhan P.K., Dubey G., Singh G., Jain D., Upadhyay S.K., Dwivedi P., Khoo K.S. (2024). Recent advancement of nano-biochar for the remediation of heavy metals and emerging contaminants: Mechanism, adsorption kinetic model, plant growth and development. Environ. Res..

[2] Liu L., Liu C., Fu R., Nie F., Zuo W., Tian Y., Zhang J. (2024). Full-chain analysis on emerging contaminants in soil: Source, migration and remediation. Chemosphere.

[3] Banica A.L., Radulescu C., Dulama I.D., Bucurica I.A., Stirbescu R.M., Stanescu S.G. (2024). Microplastic debris in yogurt: Occurrence, characterization, and implications for human health. J. Sci. Arts.

[4] Bai R., Fan R., Xie C., Liu Q., Liu Q., Yan C., Cui J., He W. (2023). Microplastics are overestimated due to poor quality control of reagents. J. Hazard. Mater..

[5] Sharma A., Gupta S., Shrivas K., Kant T. (2024). Progress in Analytical Methods for Monitoring of Heavy Metals and Metalloid in Milk and Global Health Risk Assessment. J. Food Compos. Anal..

[6] Permigiani I.S., Vallejo N.K., Hasuoka P.E., Gil R.A., Romero M.C. (2024). Arsenic speciation analysis in cow’s milk and plant-based imitation milks by HPLC-ICP-MS. J. Food Compos. Anal..

[7] Guo X., Ji X., Liu Z., Feng Z., Zhang Z., Du S., Li X., Ma J., Sun Z. (2024). Complex impact of metals on the fate of disinfection by-products in drinking water pipelines: A systematic review. Water Res..

[8] Banica A.L., Bucur R.M., Dulama I.D., Bucurica I.A., Stirbescu R.M., Radulescu C. (2023). Assessment of microplastics in personal care products by microscopic methods and vibrational spectroscopy. Sci. Study Res. Chem. Chemic. Eng. Biotechnol. Food Ind..

[9] Seetha B.S., Bhandi M.M., Shaikh A., Matta S., Mudiam M.K.R.M. (2024). Natural hydrophobic deep eutectic solvent based dispersive liquid-liquid microextraction followed by liquid chromatography with tandem mass spectrometry analysis of multi-class metabolites of pesticides, phthalates, and polycyclic aromatic hydrocarbons in animal-originated foods. Microchem. J..

[10] Yurtsever M., Cuvelek M.A. (2024). Abundance of microplastics in the agro-industrial product beet sugar; food or plastifood. Process Saf. Environ. Prot..

[11] Agus B.A.P., Rajentran K., Selamat J., Lestari S.D., Umar N.B., Hussain N. (2023). Determination of 16 EPA PAHs in food using gas and liquid chromatography. J. Food Compos. Anal..

[12] International Agency for Research on Cancer (IARC) (2010). IARC Monographs on the Evaluation of Carcinogenic Risks to Humans Volume 92: Some Non-heterocyclic Polycyclic Aromatic Hydrocarbons and Some Related Exposures.

[13] Radulescu C., Dulama I.D., Banica A.L., Bucurica I.A., Stirbescu R.M., Gorghiu L.M. (2023). Fast Method of Isolating Microplastics from Milk, Yoghurt, Sour Cream and. Butter. Patent.

[14] Radulescu C., Dulama I.D., Banica A.L., Bucurica I.A., Stirbescu R.M., Gorghiu L.M. (2024). Rapid Method for Isolation of Microplastics from Milk, Yogurt, Sour Cream, and. Butter. Patent.

[15] (2015). Cleanrooms and Associated Controlled Environments—Part 1. Classification of air Cleanliness by Particle Concentration.

[16] Girelli A.M., Sperati D., Tarola A.M. (2014). Determination of polycyclic aromatic hydrocarbons in Italian milk by HPLC with fluorescence detection. Food Addit. Contam. Part A.

[17] Kilic-Altun S., Aydemir M.E. (2021). Determination of some minerals and heavy metal levels in Urfa cheese and cow’s milk. Food Health.

[18] Tomlinson D.L., Wilson J.G., Harris C.R., Jeffrey D.W. (1980). Problems in the assessment of heavy-metal levels in estuaries and the formation of a pollution index. Helgol. Mar. Res..

[19] Fadare O.O., Okffo E.D., Olasehinde E.F. (2021). Microparticles and microplastics contamination in African table salts. Mar. Pollut. Bull..

[20] Lin Q., Zhao S., Pang L., Sun C., Chen L., Li F. (2022). Potential risk of microplastics in processed foods: Preliminary risk assessment concerning polymer types, abundance, and human exposure of microplastics. Ecotoxicol. Environ. Saf..

[21] Khan S., Farooq R., Shahbaz S., Khan M.A., Sadique M. (2009). Health risk assessment of heavy metals for population via consumption of vegetables. World Appl. Sci. J..

[22] Linkuku A.S., Obuseng G. Health risk assessment of heavy metals via dietary intake of vegetables irrigated with treated wastewater around Gaborone, Botswana. Proceedings of the International Conference on Plant, Marine and Environmental Sciences (PMES–2015).

[23] Pehoiu G., Murarescu O., Radulescu C., Dulama I.D., Teodorescu S., Stirbescu R.M., Bucurica I.A., Stanescu S.G. (2020). Heavy metals accumulation and translocation in native plants grown on tailing dumps and human health risk. Plant Sci..

[24] Atique Ullah A.K.M., Maksud M.A., Khan S.R., Lutfa L.N., Quraishi S.B. (2017). Dietary intake of heavy metals from eight highly consumed species of cultured fish and possible human health risk implications in Bangladesh. Toxicol. Rep..

[25] Kohn H.F., Hubert L.J. (2015). Hierarchical cluster analysis. Wiley StatsRef: Statistics Reference Online (WSR).

[26] Granato D., Santos J.S., Escher G.B., Ferreira B.L., Maggio R.M. (2018). Use of principal component analysis (PCA) and hierarchical cluster analysis (HCA) for multivariate association between Bioactive compounds and functional properties in foods: A critical perspective. Trends Food Sci. Technol..

[27] Kutralam-Muniasamy G., Perez-Guevara F., Elizalde-Martinez I., Shruti V.C. (2020). Branded milks—Are they immune from microplastics contamination?. Sci. Total Environ..

[28] Basaran B., Ozcifci Z., Akcay H.T., Aytan U. (2023). Microplastics in branded milk: Dietary exposure and risk assessment. J. Food Compos. Anal..

[29] Buyukunal S.K., Zipak S.R., Muratoglu K. (2023). Microplastics in a Traditional Turkish Dairy Product: Ayran. Pol. J. Food Nutr. Sci..

[30] Diaz-Basantes M.F., Conesa J.A., Fullana A. (2020). Microplastics in honey, beer, milk and refreshments in Ecuador as emerging contaminants. Sustainability.

[31] Da Costa Filho P.A., Andrey D., Eriksen B., Peixoto R.P., Carreres B.M., Ambühl M.E., Descarrega J.B., Dubascoux S., Zbinden P., Panchaud A. (2021). Detection and characterization of small-sized microplastics (≥ 5 μm) in milk products. Sci. Rep..

[32] Zarafu I., Matei L., Bleotu C., Ionita P., Tatibouët A., Paun A., Nicolau I., Hanganu A., Limban C., Nuta D.C. (2020). Synthesis, Characterization, and Biologic Activity of New Acyl Hydrazides and 1,3,4-Oxadiazole Derivatives. Molecules.

[33] Mecozzi M., Pietroletti M., Monakhova Y.B. (2016). FTIR spectroscopy supported by statistical techniques for the structural characterisation of plastic debris in the marine environment: Application to monitoring studies. Mar. Poll. Bull..

[34] Jung M.R., Horgen F.D., Orski S.V., Rodriguez V., Beers K.L., Balazs G.H., Jones T.T., Work T.M., Brignac K.C., Royer S.J. (2018). Validation of ATR-FTIR to identify polymers of plastic marine debris, including those ingested by marine organisms. Mar. Poll. Bull..

[35] Noda I., Dowrey A.E., Haynes J.L., Marcott C., Mark J.E. (2007). Group frequency assignments for major infrared bands observed in common synthetic polymers. Physical Properties of Polymers Handbook.

[36] Bonello G., Varrella P., Pane L. (2018). First Evaluation of Microplastic Content in Benthic Filter-feeders of the Gulf of La Spezia (Ligurian Sea). J. Aquat. Food Prod. Technol..

[37] Torres-Moreno C., Puente-DelaCruz L., Codling G., Villa A.L., Cabo M., Klanova J., Johnson-Restrepo J. (2022). Polycyclic aromatic hydrocarbons (PAHs) in human breast milk from Colombia: Spatial occurrence, sources and probabilistic risk assessment. Environ. Res..

[38] Armbruster D.A., Pry T. (2008). Limit of Blank, Limit of Detection and Limit of Quantitation. Clin. Biochem. Rev..

[39] Huber L. (2007). Validation and Qualification in Analytical Laboratories.

[40] Ali M., Xu D., Yang X., Hu J. (2024). Microplastics and PAHs mixed contamination: An in-depth review on the sources, co-occurrence, and fate in marine ecosystems. Water Res..

[41] Lee J., Jeong S. (2023). Approach to an answer to “How dangerous microplastics are to the human body”: A systematic review of the quantification of MPs and simultaneously exposed chemicals. J. Hazard. Mater..

[42] Leslie H.A., van Velzen M.J.M., Brandsma S.H., Vethaak A.D., Garcia-Vallejo J.J., Lamoree M.H. (2022). Discovery and quantification of plastic particle pollution in human blood. Environ. Int..

[43] Ragusa A., Svelato A., Santacroce C., Catalano P., Notarstefano V., Carnevali O., Papa F., Rongioletti M.C.A., Baiocco F., Draghi S. (2021). Plasticenta: First evidence of microplastics in human placenta. Environ. Int..

[44] Zhu L., Zhu J., Zuo R., Xu Q., Qian Y., An L. (2023). Identification of microplastics in human placenta using laser direct infrared spectroscopy. Sci. Total Environ..

[45] Ragusa A., Notarstefano V., Svelato A., Belloni A., Gioacchini G., Blondeel C., Zucchelli E., De Luca C., D’Avino S., Gulotta A. (2022). Raman Microspectroscopy Detection and Characterisation of Microplastics in Human Breastmilk. Polymers.

