# Supplementary material for: Microplastics, Polycyclic Aromatic Hydrocarbons, and Heavy Metals in Milk: Analyses and Induced Health Risk Assessment
Source: Foods. 2024 Sep 26;13(19):3069. doi: 10.3390/foods13193069 (PMC11476328; doi:10.3390/foods13193069)
Supplement: Supplementary file 1 [file foods-13-03069-s001.zip › foods-3168577-supplementary.pdf]

Supplementary material

Microplastics, Polycyclic Aromatic Hydrocarbons, and Heavy Metals in Milk: Analyses and Induced Health Risk Assessment

Andreea Laura Banica [1,2], Cristiana Radulescu [2,3,4,*], Ioana Daniela Dulama [1], Ioan Alin Bucurica [1], Raluca Maria Stirbescu [1] and Sorina Geanina Stanescu [1]

[1] Institute of Multidisciplinary Research for Science and Technology, Valahia University of Targoviste, 13 Sinaia Alley, 130004 Targoviste, Romania; banica.andreea@icstm.ro (A.L.B.); dulama.ioana@icstm.ro (I.D.D.); bucurica_alin@icstm.ro (I.A.B.); stirbescu.raluca@icstm.ro (R.M.S.); geanina.stanescu@icstm.ro (S.G.S.)

[2] Doctoral School Chemical Engineering and Biotechnology, National University of Science and Technology Politehnica of Bucharest, 313 Splaiul Independent,ei, 060042 Bucharest, Romania

[3] Faculty of Sciences and Arts, Valahia University of Targoviste, 13 Sinaia Alley, 130004 Targoviste, Romania

[4] Academy of Romanian Scientists, 3 Ilfov, 050044 Bucharest, Romania

* Correspondence: cristiana.radulescu@valahia.ro

L1

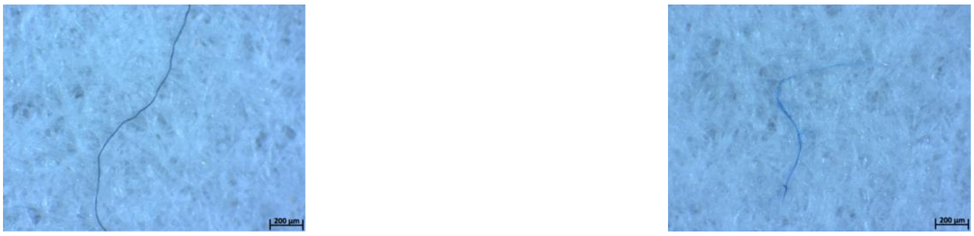

L2

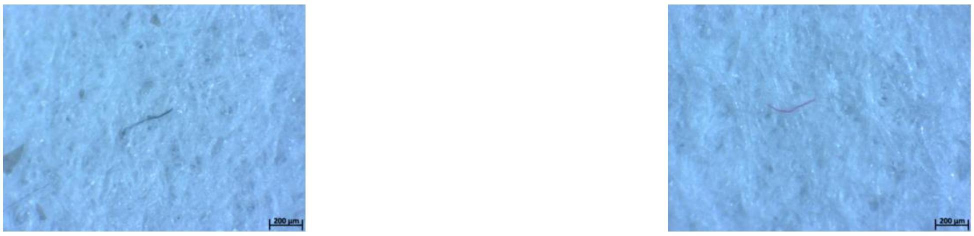

L3

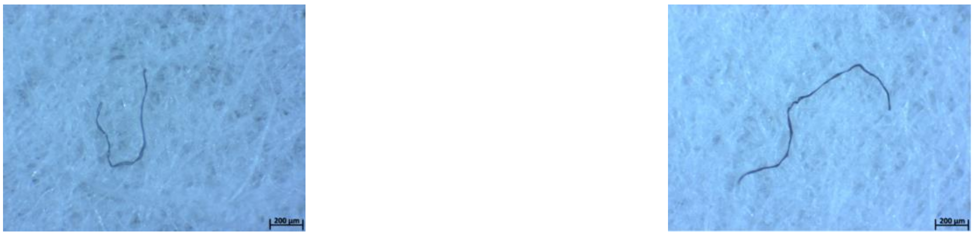

L4

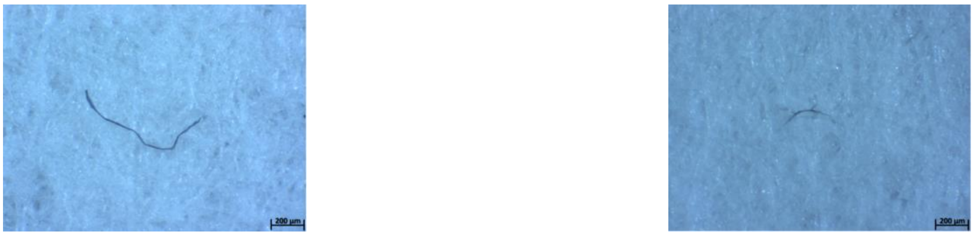

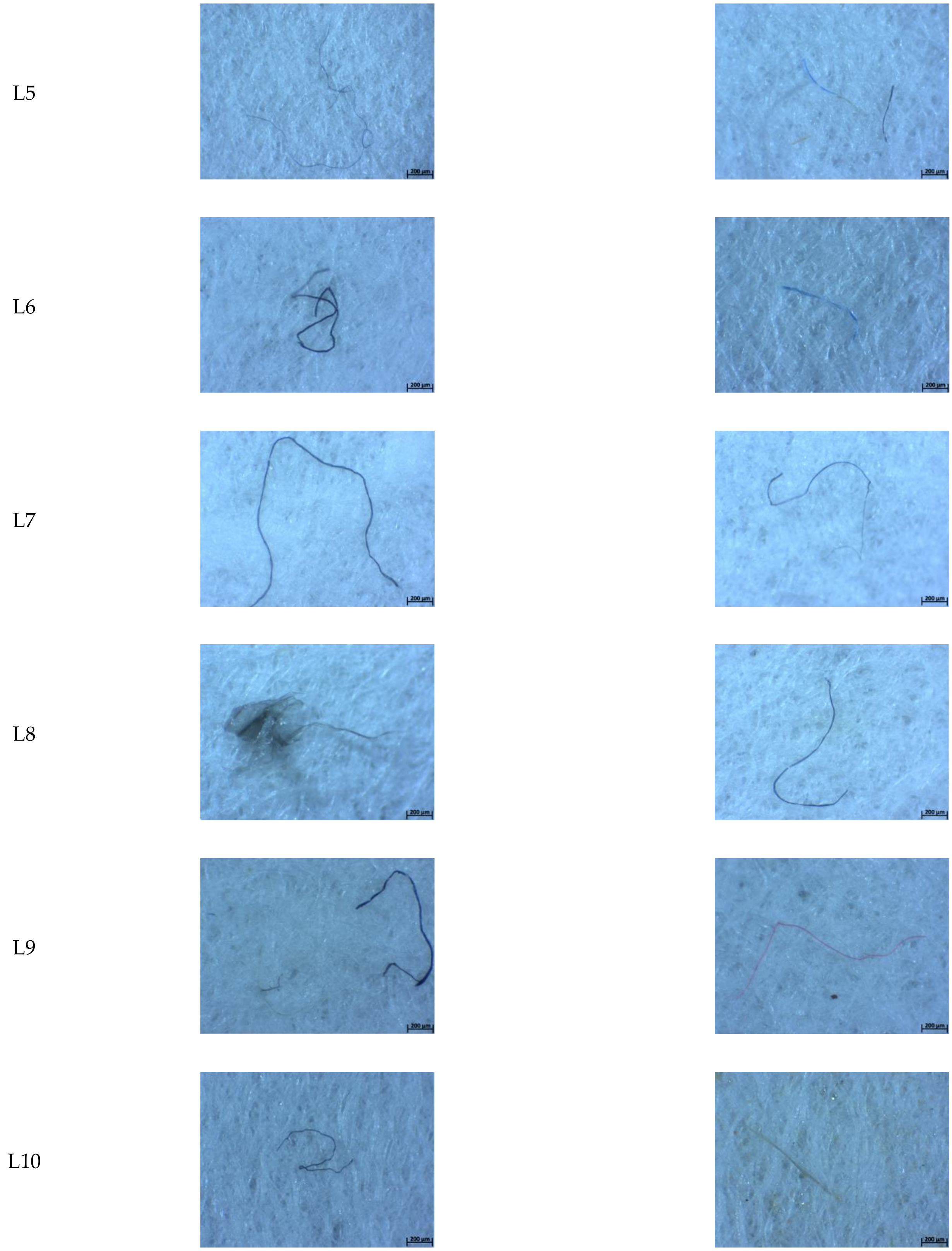

Figure S1. Optical microscopy images of representative MPs identified in conventional milk.

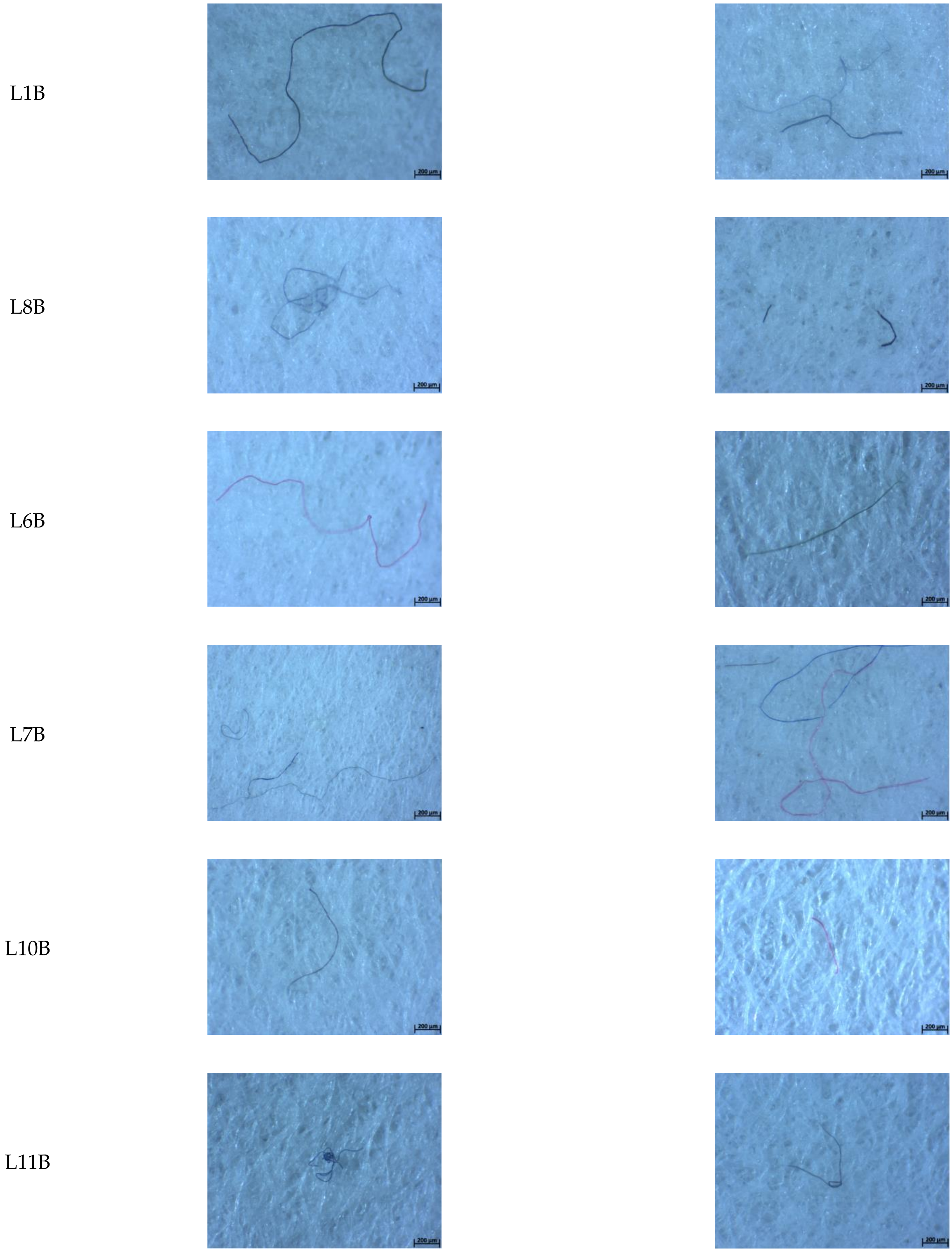

Figure S2. Optical microscopy images of representative Mps identified in organic milk.

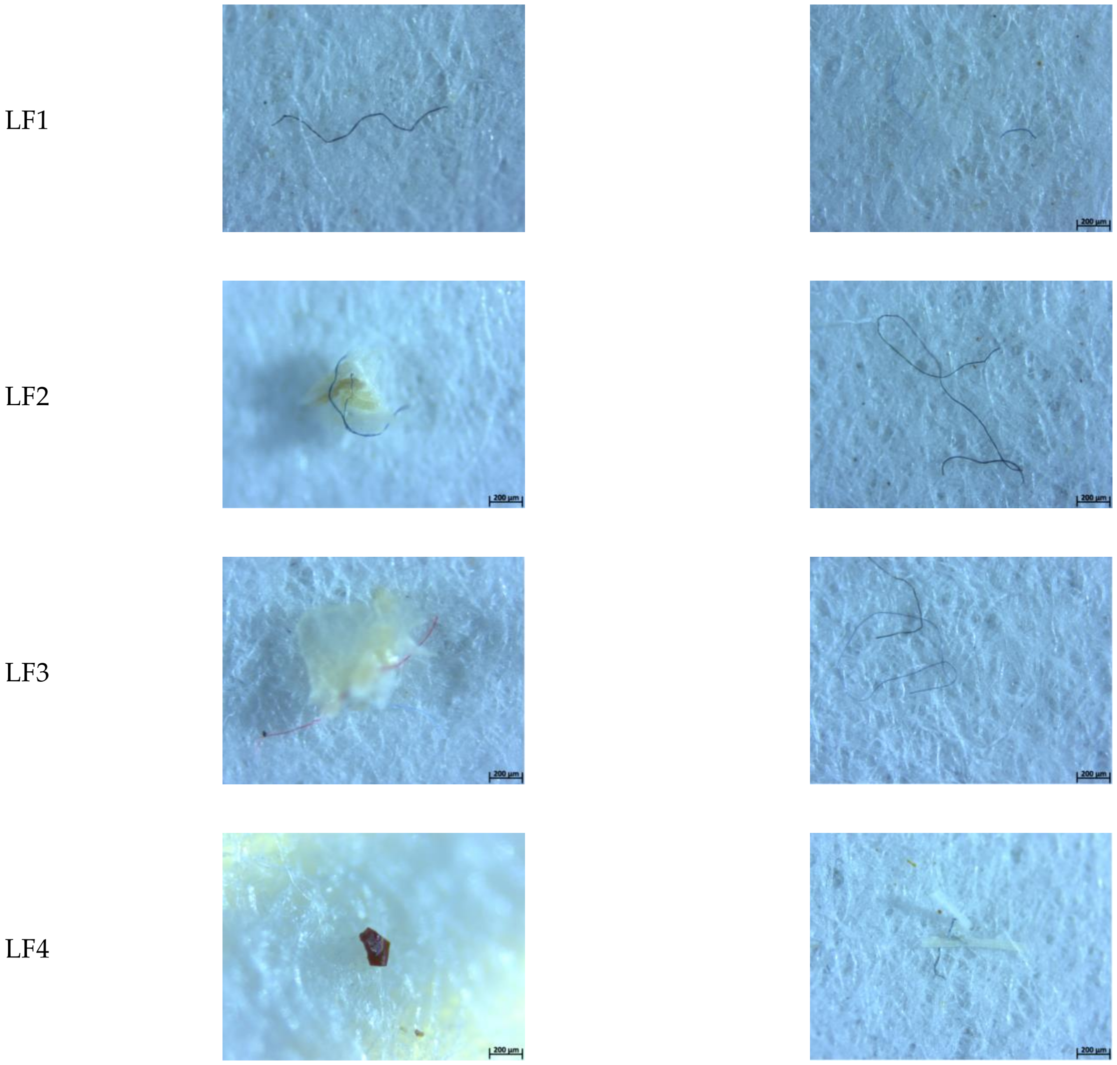

Figure S3. Optical microscopy images of representative Mps identified in raw milk.

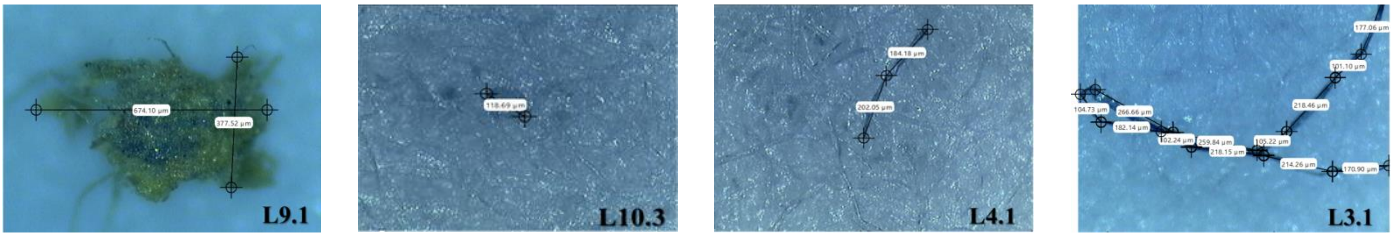

Figure S4. Measurement sequence of microparticles identified in conventional milk samples.

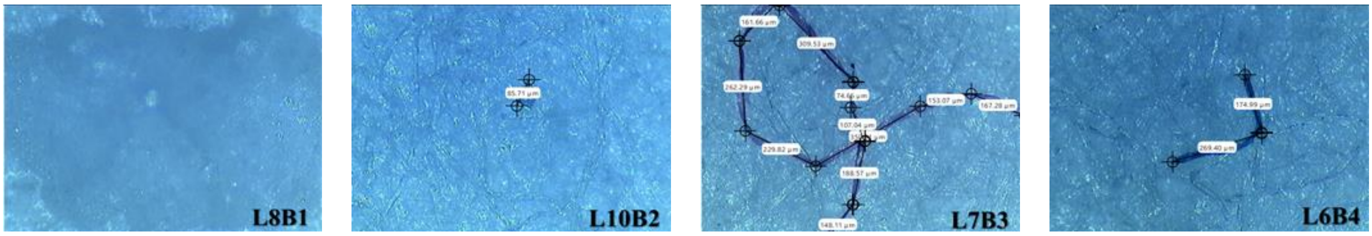

Figure S5. Measurement sequence of microparticles identified in organic milk samples.

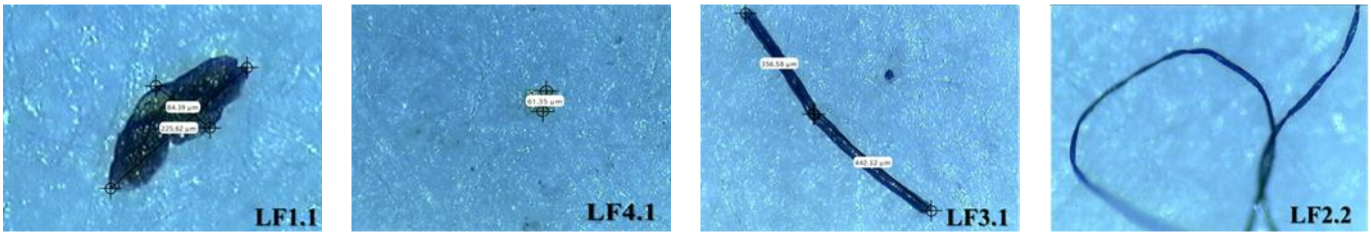

Figure S6. Measurement sequence of microparticles identified in raw milk samples.

Table S1. General presentation of conventional milk samples.

Sample code	Nutritional value / 100 g milk						Fat content [%]	Packaging type
	Energy value		Saturated fatty acids [g]	Carbohydrates [g]	Protein [g]	Salt [g]		
	[kJ]	[kcal]						
L1	190	45	0.9	4.7	3.2	0.12	1.5	Tetra Pak
L2	207	49	1.2	4.8	3.0	0.11	1.5	Tetra Pak
L3	207	49	1.2	4.8	3.0	0.11	1.5	Plastic
L4	185	44	1.0	4.5	3.1	0.10	1.5	Tetra Pak
L5	185	44	1.0	4.5	3.1	0.10	1.5	Plastic
L6	185	44	1.0	4.5	3.1	0.10	1.5	Tetra Pak
L7	185	44	1.0	4.5	3.1	0.00	1.5	Tetra Pak
L8	256	64	2.5	3.8	3.2	0.10	3.8	Glass
L9	138	45	1.0	4.5	1.3	0.60	1.5	Tetra Pak
L10	186	44	0.9	4.5	3.2	0.06	1.5	Tetra Pak

Table S2. General presentation of organic milk samples.

Sample code	Nutritional value / 100 g milk						Fat content [%]	Packaging type
	Energy value		Saturated fatty acids [g]	Carbohydrates [g]	Protein [g]	Salt [g]		
	[kJ]	[kcal]						
L1B	198	47	0.9	4.9	3.5	0.13	1.5	Tetra Pak
L6B	269	64	1.0	4.5	3.2	0.10	3.7	Tetra Pak
L7B	185	44	0.9	4.5	3.1	0.10	1.5	Tetra Pak
L8B	297	71	2.0	4.0	3.7	0.00	4.1	Glass
L10B	270	65	2.3	4.5	3.1	0.06	1.5	Tetra Pak
L11B	187	45	1.0	4.5	3.0	0.10	1.5	Tetra Pak

Table S3. Identification of MPs according to the spectra library of OPUS software v.7.5 (conventional milk samples).

Sample Code	Micro-FTIR images	Cotton	Poly(methyl methacrylate)	Polyamides (Nylon)	Polyurethane (Elastane)	Cellulose	Polyester	Flax	Polyethylene	Composition of microplastics [%]	Morphology	
											Shape	Size (LxW) [μm]
L1.1	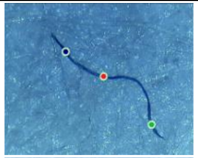	✓		✓	✓	✓				mixture 33:16:2:49	Fiber	801.35 (L)
L2.1	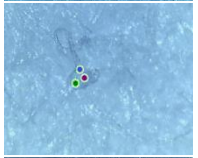								✓	100%	Oval	168.11x92.45
L2.2	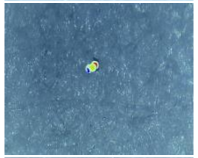	✓	✓			✓				mixture 60:20:20	Fragment	76.00 (L)
L2.3	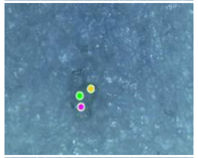				✓					100%	Fragment	215.36x156.91
L2.4	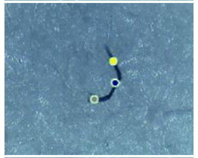						✓			100%	Fiber	384.74 (L)
L4.1	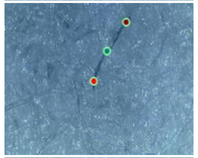	✓	✓	✓		✓				mixture 70:6:11:13	Fiber	368.23 (L)
L4.2	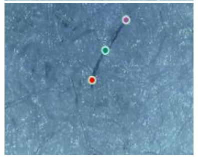	✓	✓	✓		✓				mixture 70:6:11:13	Fiber	432.43 (L)

Sample Code	Micro-FTIR images	Cotton	Poly(methyl methacrylate)	Polyamides (Nylon)	Polyurethane (Elastane)	Cellulose	Polyester	Flax	Polyethylene	Composition of microplastics [%]	Morphology	
											Shape	Size (LxW) [μm]
L5.1	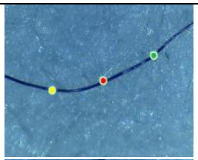	✓	✓			✓				mixture 60:20:20	Fiber	>1021.58 (L)
L5.2	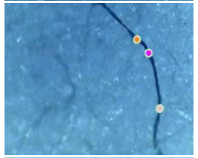	✓					✓			mixture 70:30	Fiber	>817.71(L)
L7.1	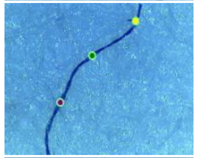	✓	✓	✓		✓				mixture 70:6:11:13	Fiber	882.26 (L)
L7.2	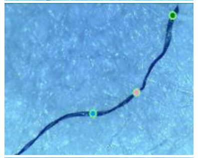	✓	✓					✓		mixture 64:15:21	Fiber	1129.28 (L)
L7.3	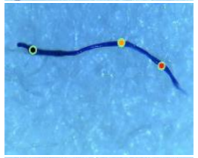	✓			✓					mixture 95:5	Fiber	895.58 (L)
L8.1	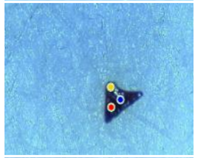	✓	✓	✓		✓				mixture 70:6:11:13	Fragment (triangle)	22.54x175.34
L8.2	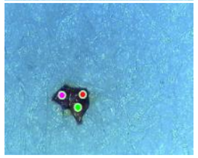	✓	✓							mixture 50:50	Fragment (irregular)	174.28x166.37

Sample Code	Micro-FTIR images	Cotton	Poly(methyl methacrylate)	Polyamides (Nylon)	Polyurethane (Elastane)	Cellulose	Polyester	Flax	Polyethylene	Composition of microplastics [%]	Morphology	
											Shape	Size (LxW) [μm]
L8.3	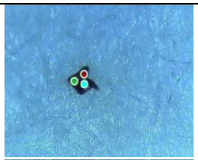	✓	✓					✓		mixture 64:15:21	Fragment (square)	111.25x100.87
L8.4	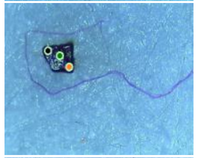	✓	✓							mixture 79:21	Fragment (square)	151.07x142.96
L8.5	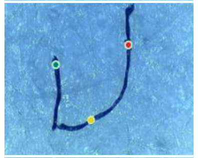	✓	✓	✓		✓				mixture 70:6:11:13	Fiber	1081.33 (L)
L8.6	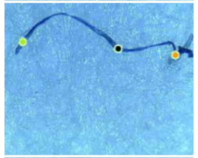	✓	✓							mixture 60:40	Fiber	>1036.50 (L)
L8.7	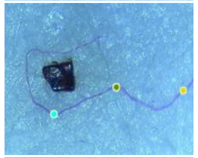	✓	✓							mixture 60:40	Fiber	>1507.22 (L)
L9.1	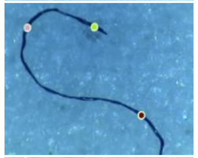	✓	✓					✓		mixture 64:15:21	Fiber	>1422.21 (L)
L9.2	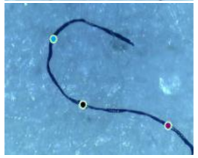	✓	✓	✓		✓				mixture 70:6:11:13	Fiber	>1424.71 (L)

Sample Code	Micro-FTIR images	Cotton	Poly(methyl methacrylate)	Polyamides (Nylon)	Polyurethane (Elastane)	Cellulose	Polyester	Flax	Polyethylene	Composition of microplastics [%]	Morphology	
											Shape	Size (LxW) [μm]
L9.3	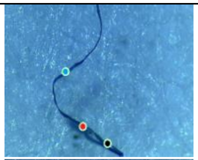	✓	✓	✓		✓				mixture 70:6:11:13	Fiber	>970.15 (L)
L10.1	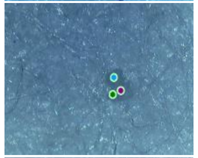	✓		✓						mixture 60:40	Fragment (irregular)	145.18x121.91
L10.2	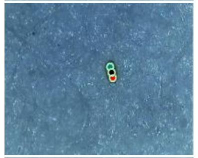	✓			✓					mixture 95:5	Fragment (oval)	110.05 (L)
L10.3	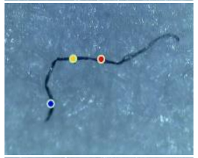			✓		✓	✓			mixture 33.33:33.33:33.33	Fiber	883.79 (L)
L10.4	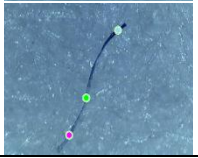	✓			✓					mixture 95:5	Fiber	662.68 (L)

Table S4. Identification of MPs according to the spectra library of OPUS software v.7.5 (organic milk samples).

Sample Code	Micro-FTIR images	Cotton	Poly(methyl methacrylate)	Polyamides (Nylon)	Polyurethane (Elastane)	Cellulose	Polyester	Composition of microplastics [%]	Morfology	
									Shape	Size (LxW) [μm]
L1B1	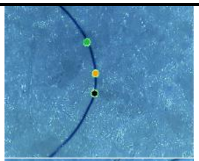	✓	✓					mixture 60:40	Fiber	851.61 (L)
L1B2	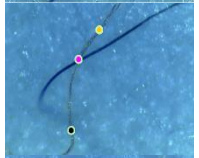	✓		✓	✓	✓		mixture 33:16:2:49	Fiber	-
L8B1	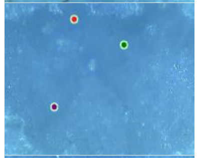	✓		✓		✓		mixture 65:25:10	Fragment (irregular)	-
L8B2	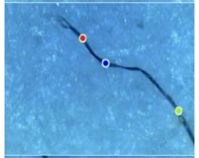	✓					✓	mixture 85:15	Fiber	>904.51 (L)
L8B3	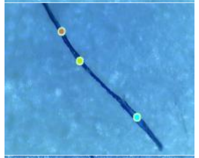	✓	✓	✓		✓		mixture 70:6:11:13	Fiber	>873.77 (L)
L8B4	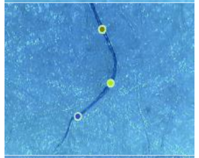	✓	✓	✓		✓		mixture 70:6:11:13	Fiber	815.32 (L)
L8B5	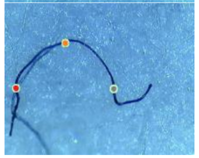	✓	✓					mixture 60:40	Fiber	>1146.11 (L)

Sample Code	Micro-FTIR images	Cotton	Poly(methyl methacrylate)	Polyamides (Nylon)	Polyurethane (Elastane)	Cellulose	Polyester	Composition of microplastics [%]	Morfology	
									Shape	Size (LxW) [μm]
L8B6	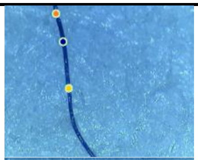	✓		✓	✓	✓		mixture 33:16:2:49	Fiber	>765.13 (L)
L6B1	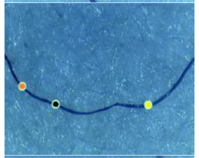	✓		✓		✓		mixture 65:25:10	Fiber	1158.56 (L)
L7B1	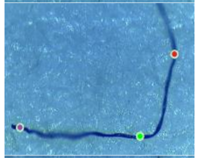	✓	✓	✓		✓		mixture 70:6:11:13	Fiber	625.31 (L)
L7B2	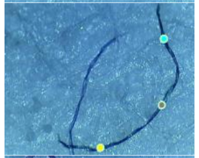	✓		✓	✓	✓		mixture 33:16:2:49	Fiber	1056.73 (L)
L7B3	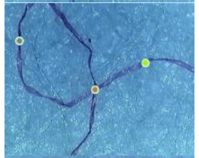	✓	✓					mixture 50:50	Fiber	2152.24 (L)
L7B4	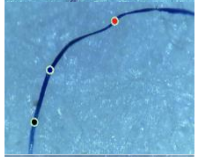	✓		✓		✓		mixture 65:25:10	Fiber	1202.08 (L)
L10B1	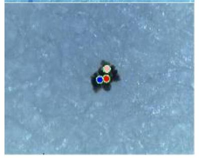	✓	✓			✓		mixture 60:20:20	Fragment (irregular)	68.05x67.83 (L)

Sample Code	Micro-FTIR images	Cotton	Poly(methyl methacrylate)	Polyamides (Nylon)	Polyurethane (Elastane)	Cellulose	Polyester	Composition of microplastics [%]	Morfology	
									Shape	Size (LxW) [μm]
L10B2	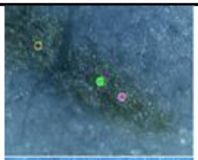	✓		✓				mixture 60:40	Fragment (irregular)	85.71x58.85 (L)
L11B1	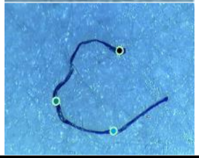	✓	✓	✓		✓		mixture 70:6:11:13	Fiber	565.98 (L)

Table S5. Identification of MPs according to the spectra library of OPUS software v.7.5 (raw milk samples).

Sample Code	Micro-FTIR images	Cotton	Poly(methyl methacrylate)	Polyamides (Nylon)	Cellulose	Flax	Composition of microplastics [%]	Morfology	
								Shape	Size (LxW) [µm]
LF1.1	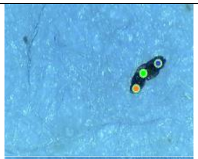	✓	✓			✓	mixture 64:15:21	Fragment (irregular)	225.62x84.39
LF1.2	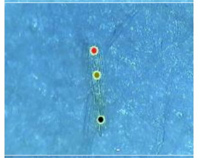	✓	✓				mixture 60:40	Fiber	391.20 (L)
LF1.3	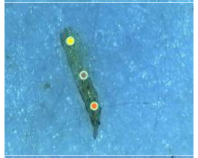	✓	✓	✓	✓		mixture 70:6:11:13	Fiber	516.39 (L)
LF2.1	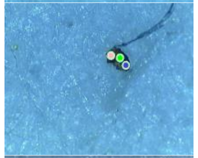	✓		✓	✓		mixture 65:25:10	Fragment (irregular)	135.22x96.35
LF2.2	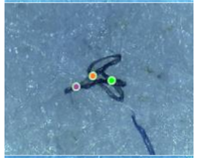	✓	✓				mixture 60:40	Fiber	-
LF2.3	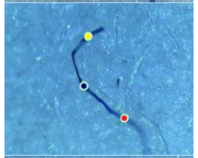	✓	✓	✓	✓		mixture 70:6:11:13	Fiber	-
LF2.4	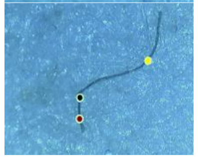	✓	✓	✓	✓		mixture 70:6:11:13	Fiber	773 (L)

Sample Code	Micro-FTIR images	Cotton	Poly(methyl methacrylate)	Polyamides (Nylon)	Cellulose	Flax	Composition of microplastics [%]	Morfology	
								Shape	Size (LxW) [µm]
LF3.1	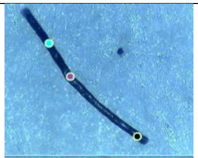	✓		✓	✓		mixture 65:25:10	Fiber	798.90 (L)
LF3.2	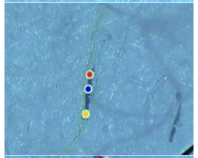	✓	✓			✓	mixture 64:15:21	Fiber	232.26 (L)
LF3.3	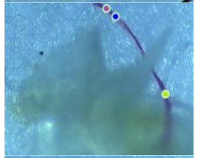	✓	✓				mixture 60:40	Fiber	-
LF3.4	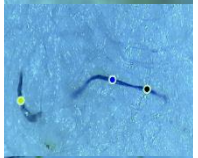	✓	✓	✓	✓		mixture 70:6:11:13	Fiber	-
LF3.5	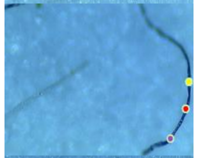	✓		✓	✓		mixture 65:25:10	Fiber	-
LF4.1	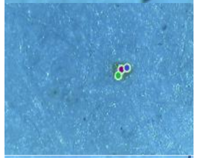	✓	✓	✓	✓		mixture 70:6:11:13	Fragment (irregular)	115.53x61.35
LF4.2	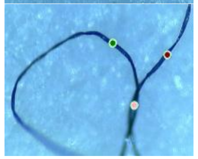	✓	✓				mixture 60:40	Fiber	-

Sample Code	Micro-FTIR images	Cotton	Poly(methyl methacrylate)	Polyamides (Nylon)	Cellulose	Flax	Composition of microplastics [%]	Morfology	
								Shape	Size (LxW) [µm]
LF4.3	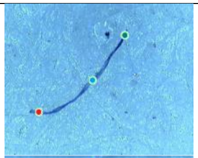	✓	✓	✓	✓		mixture<br>70:6:11:13	Fiber	618.34 (L)
LF4.4	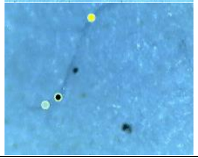	✓	✓				mixture<br>79:21	Fiber	-

Apart from FTIR spectra that non-destructive characterize the polymeric structure of MPs, high-resolution micro-FTIR imaging allows visualization and mapping of MP particles on a micrometer scale, promoting an advanced understanding of their structure. Starting from the chronological procedural order considered by the authors to be natural, optical microscopy was chosen as the first step to identify the microparticles, implicitly the characterization from the point of view of shape, and color, but also their quantification, at which point it was not possible to state which it is their chemical composition. Therefore, the identified microparticles cannot be considered microplastics and treated in other ways; this aspect will be confirmed or denied by the compositional analyses carried out by the micro-FTIR method. Therefore, a total of 96 microparticles, 51 from conventional milk, 25 from organic milk, and 20 from raw milk, were analyzed by micro-FTIR technique. Following the micro-FTIR analysis, the shape, size, and composition of the MPs were established according to the spectra library of OPUS software v.7.5, being divided into two categories (Figure S7), natural and synthetic. In the case of the natural ones, the FTIR spectra obtained and corroborated with the OPUS library showed the presence of cellulose in some microparticles and in others only cotton (Tables S3, S4, and S5). Even though cotton contains cellulose in its composition, the software did not identify them as one and the same [1-5]. In this respect, both cotton and cellulose as identified by OPUS v7.5 library were accepted and approached in this research as natural organic compounds. The presence of other compounds shows a mixture of a natural and synthetic matrix, therefore the samples were assimilated as synthetic compounds (polymer, MPs). In this respect, based on several studies [1-9], polymer type was considered acceptable in the milk sample when the match with standard spectra was greater than 70%.

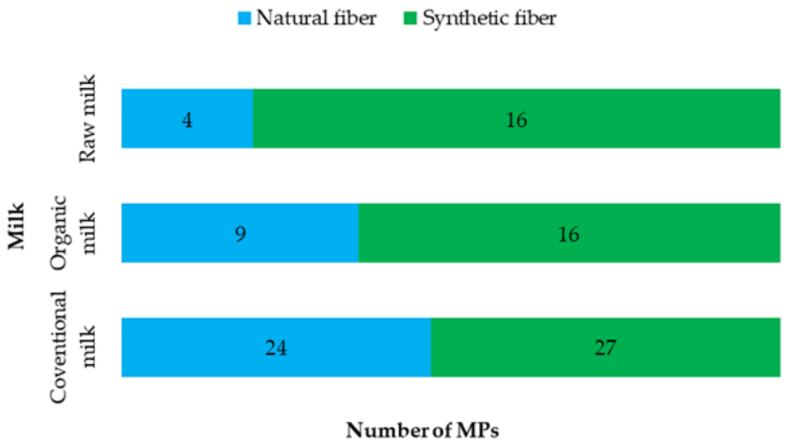

Figure S7. Natural and synthetic MPs from the analyzed milk samples.

Moreover, from the 51 microparticles analyzed from the conventional milk samples, 24 presented natural compounds (*i.e.*, cotton, cellulose), and 27 presented polymeric structures in a mixture matrix (Table S3). In the organic milk samples, 25 microparticles were analyzed of which 9 presented natural structures or mixtures and 16 polymeric mixtures (Table S4). On the other hand, for the raw milk samples, 20 microparticles were analyzed, 4 presented natural compounds and 16 presented polymeric structures in a mixture matrix (Table S5). In all three categories of milk analyzed, more polymeric microparticles, from the microplastics category, (59) than natural microparticles (37) were identified.

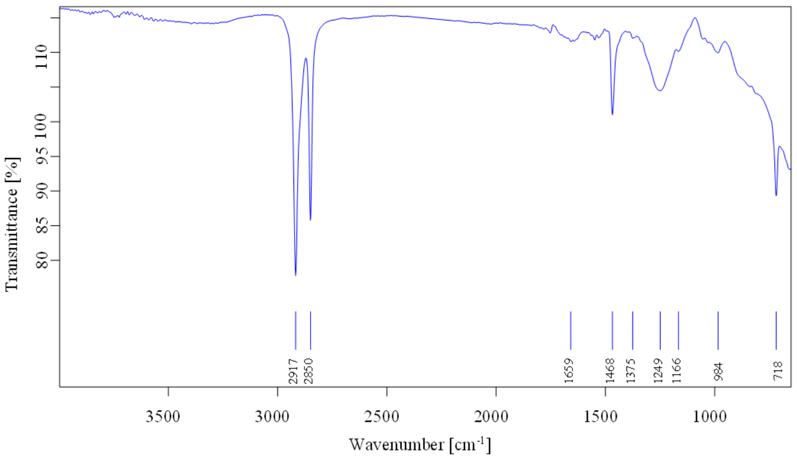

a)

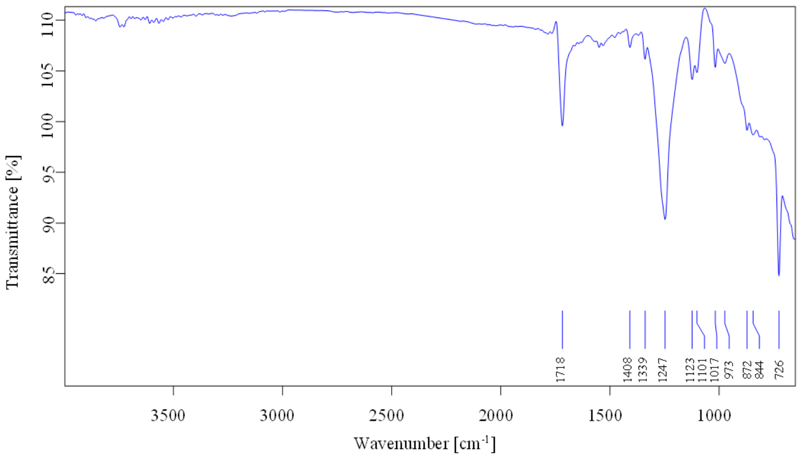

b)

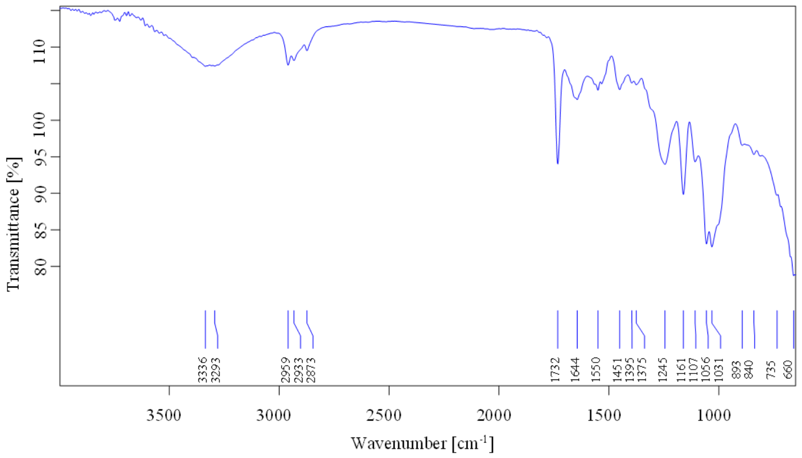

c)

Figure S8. FTIR spectra of MPs from conventional milk samples (selection): a) – polyethylene; b) – polyester; c) - polyurethane.

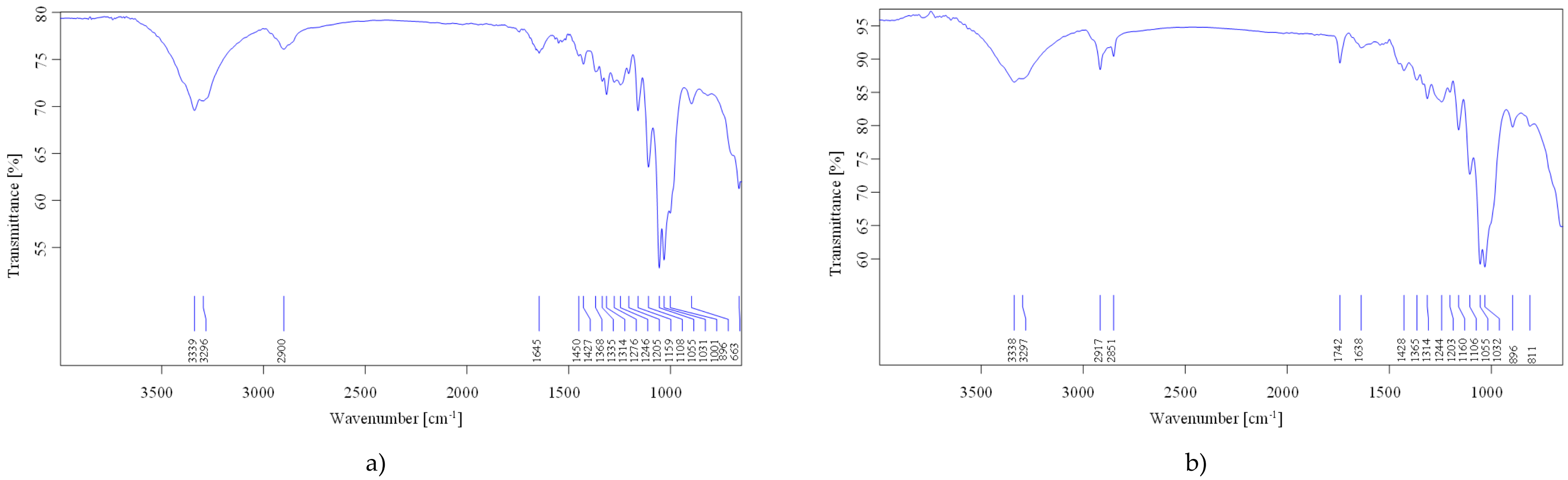

Figure S9. FTIR spectra of MPs from organic milk samples (selection): a) - mixture: cotton, nylon, and cellulose; b) - mixture: cotton and acrylic.

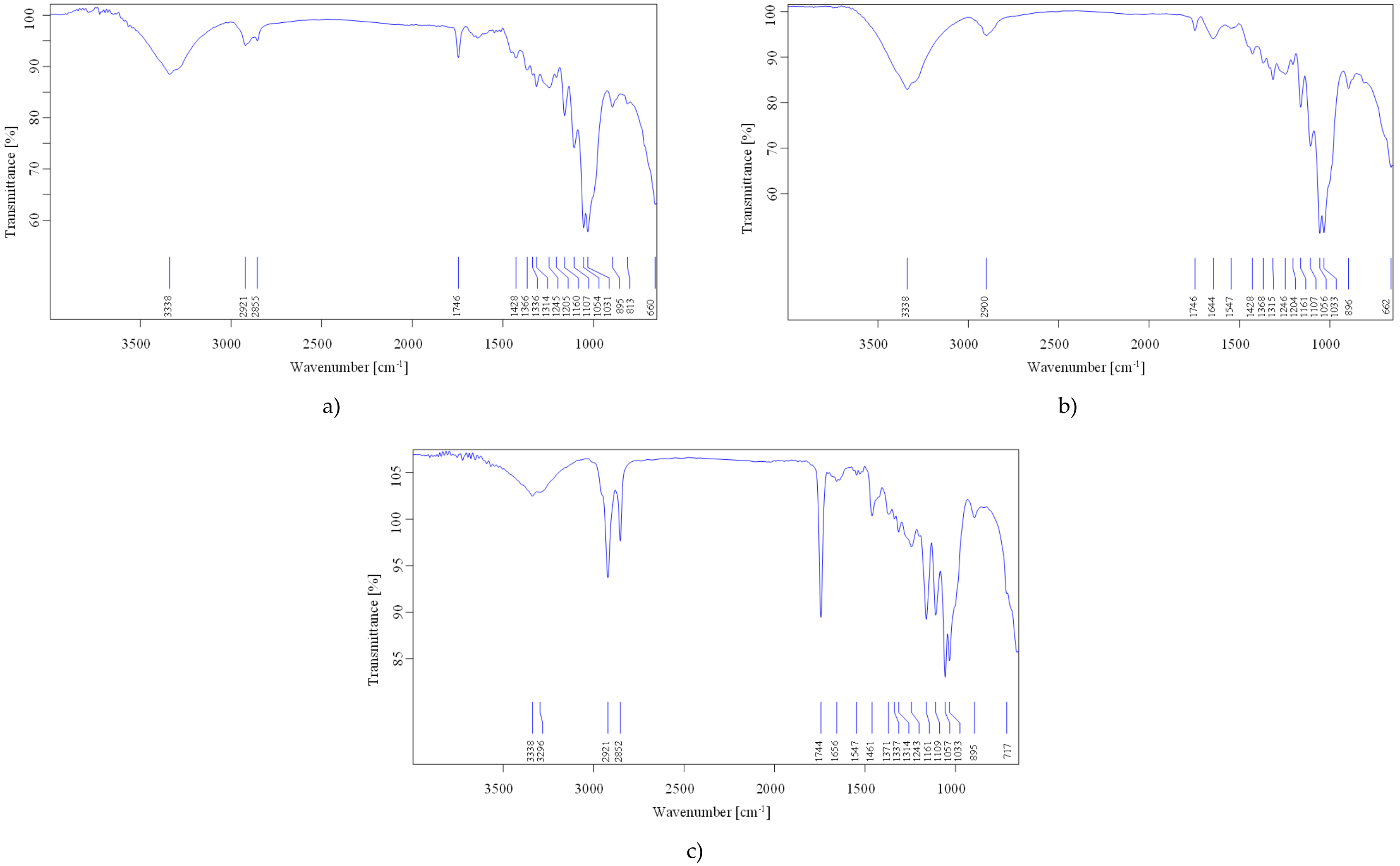

Figure S10. FTIR spectra of MPs from raw milk samples (selection): a) - mixture: cotton, arcrylic, and flax; b) - mixture: cotton, cotton, cellulose, nylon, and acrylic; c) – cotton and aclrylic.

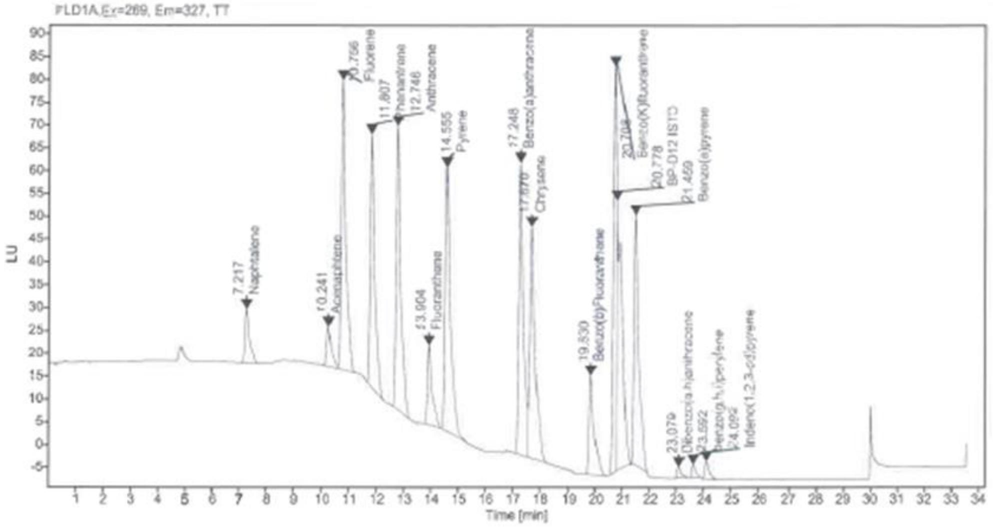

Figure S11. Calibration curve related to 16 PAHs using HPLC-FLD.

L1

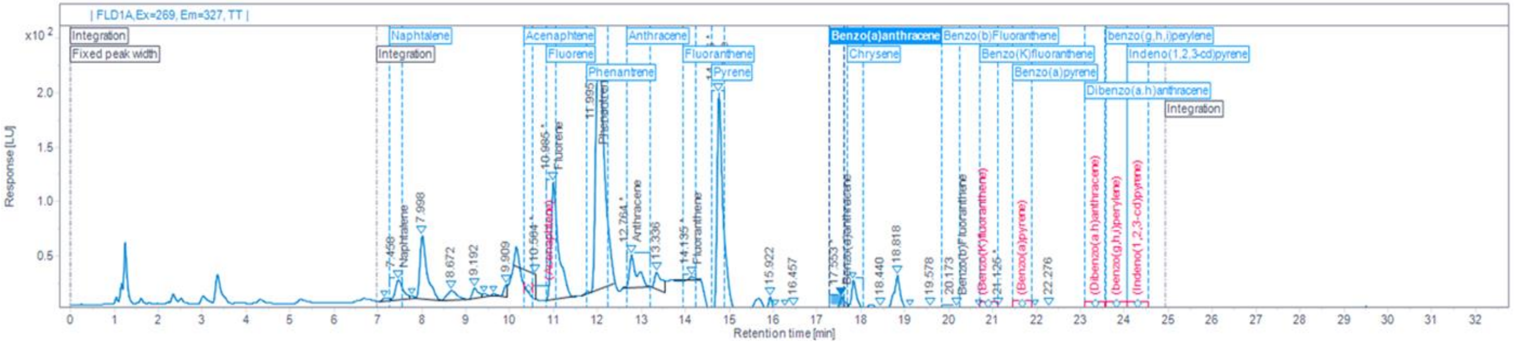
| FLD1A,Ex=269, Em=327, TT |
Integration
Fixed peak width
Naphtalene
Acenaphtene
Fluorene
Phenantrene
Anthracene
Fluoranthene
Pyrene
Benzo(a)anthracene
Chrysene
Benzo(b)Fluoranthene
Benzo(K)fluoranthene
Benzo(a)pyrene
Dibenzo(a.h)anthracene
benzo(g.h.i)perylene
Indeno(1,2,3-cd)pyrene
Response [LU]
Retention time [min]

L2

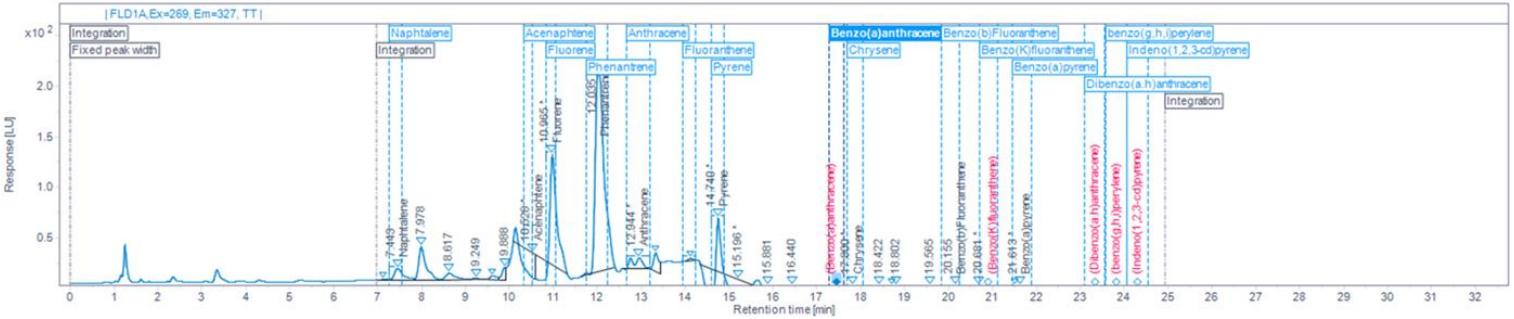
| FLD1A,Ex=269, Em=327, TT |
Integration
Fixed peak width
Naphtalene
Acenaphtene
Fluorene
Phenantrene
Anthracene
Fluoranthene
Pyrene
Benzo(a)anthracene
Chrysene
Benzo(b)Fluoranthene
Benzo(K)fluoranthene
Benzo(a)pyrene
Dibenzo(a.h)anthracene
benzo(g.h.i)perylene
Indeno(1,2,3-cd)pyrene
Response [LU]
Retention time [min]

L3

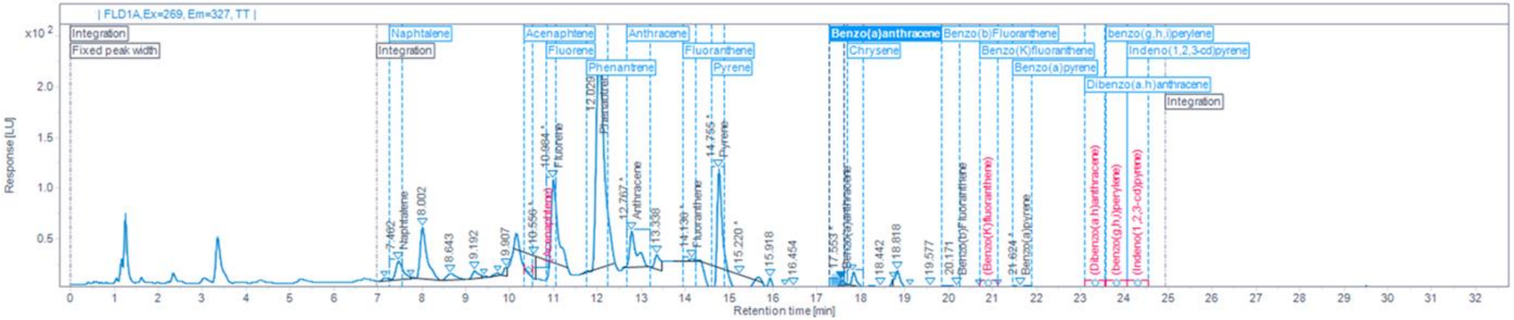
| FLD1A,Ex=269, Em=327, TT |
Integration
Fixed peak width
Naphtalene
Acenaphtene
Fluorene
Phenantrene
Anthracene
Fluoranthene
Pyrene
Benzo(a)anthracene
Chrysene
Benzo(b)Fluoranthene
Benzo(K)fluoranthene
Benzo(a)pyrene
Dibenzo(a.h)anthracene
benzo(g.h.i)perylene
Indeno(1,2,3-cd)pyrene
Response [LU]
Retention time [min]

L4

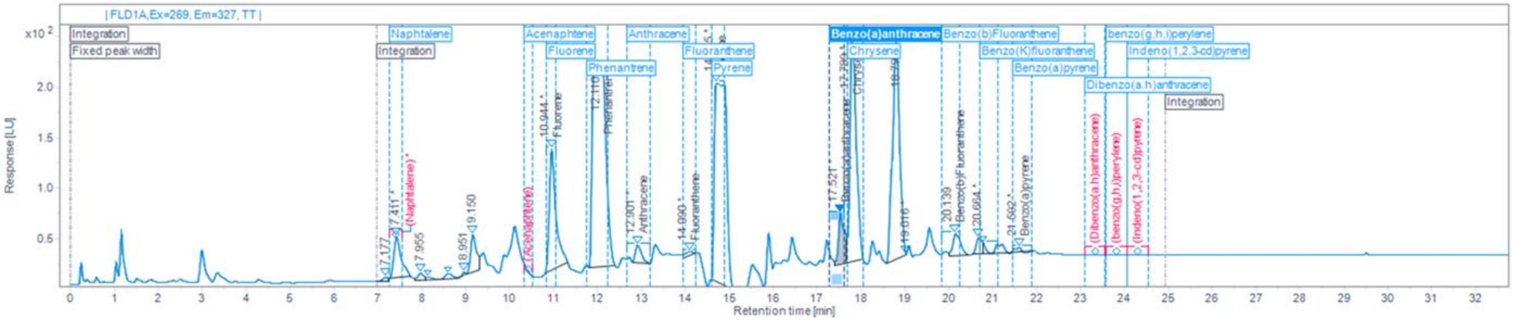

| FLD1A,Ex=269, Em=327, TT |
x10 2
Integration
Fixed peak width
Naphtalene
Acenaphtene
Fluorene
Phenantrene
Anthracene
Fluoranthene
Pyrene
Benzo(a)anthracene
Chrysene
Benzo(b)Fluoranthene
Benzo(K)fluoranthene
Benzo(a)pyrene
benzo(g,h,i)perylene
Indeno(1,2,3-cd)pyrene
Dibenzo(a,h)anthracene
Response [LU]
Retention time [min]

L5

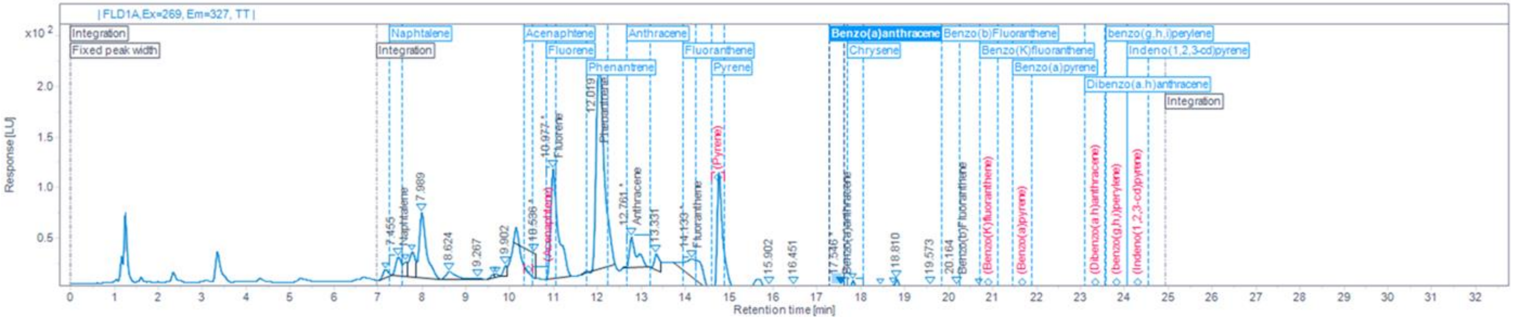

| FLD1A,Ex=269, Em=327, TT |
x10 2
Integration
Fixed peak width
Naphtalene
Acenaphtene
Fluorene
Phenantrene
Anthracene
Fluoranthene
Pyrene
Benzo(a)anthracene
Chrysene
Benzo(b)Fluoranthene
Benzo(K)fluoranthene
Benzo(a)pyrene
benzo(g,h,i)perylene
Indeno(1,2,3-cd)pyrene
Dibenzo(a,h)anthracene
Response [LU]
Retention time [min]

L6

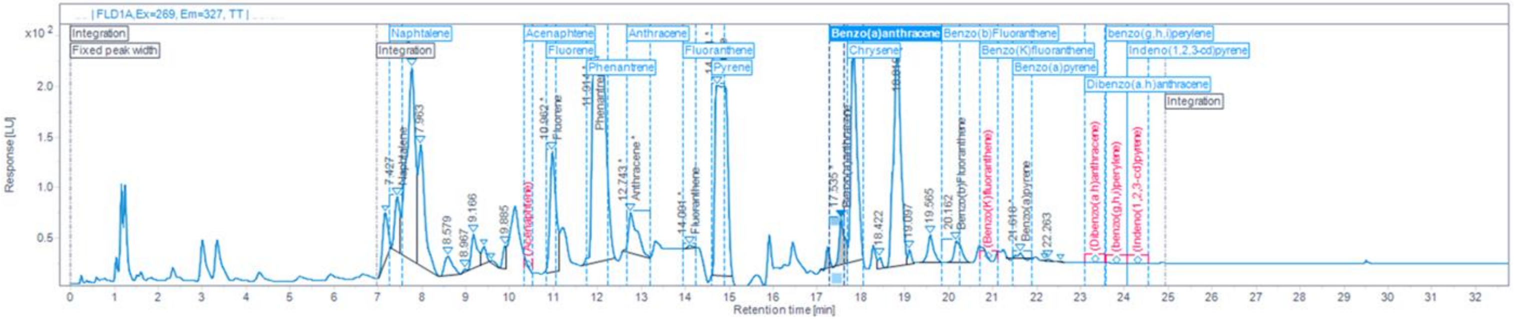

| FLD1A,Ex=269, Em=327, TT |
x10 2
Integration
Fixed peak width
Naphtalene
Acenaphtene
Fluorene
Phenantrene
Anthracene
Fluoranthene
Pyrene
Benzo(a)anthracene
Chrysene
Benzo(b)Fluoranthene
Benzo(K)fluoranthene
Benzo(a)pyrene
benzo(g,h,i)perylene
Indeno(1,2,3-cd)pyrene
Dibenzo(a,h)anthracene
Response [LU]
Retention time [min]

L7

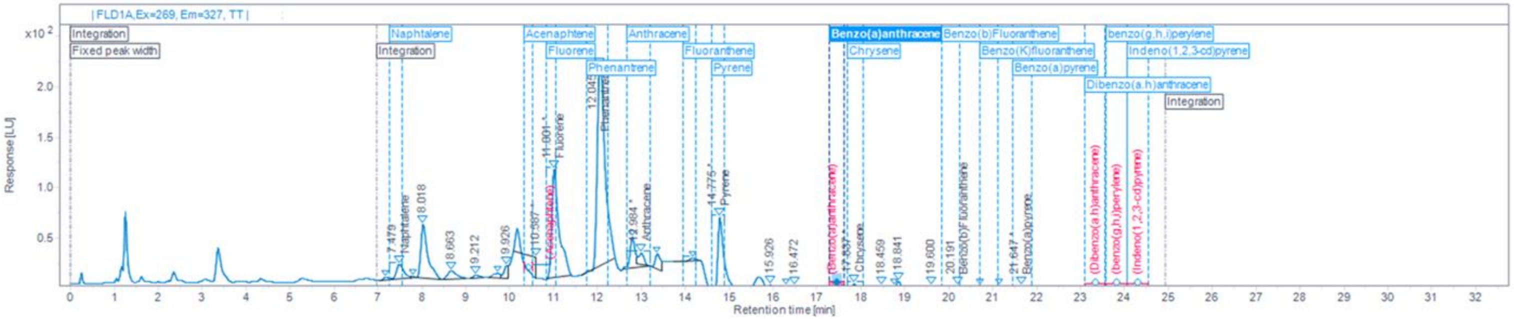
FLD1A,Ex=269, Em=327, TT
Response [LU]
x10 2
Retention time [min]
Integration
Fixed peak width
Naphtalene
Acenaphtene
Fluorene
Phenantrene
Anthracene
Fluoranthene
Pyrene
Benzo(a)anthracene
Chrysene
Benzo(b)Fluoranthene
Benzo(K)fluoranthene
Benzo(a)pyrene
Dibenzo(a,h)anthracene
benzo(g,h,i)perylene
Indeno(1,2,3-cd)pyrene

L8

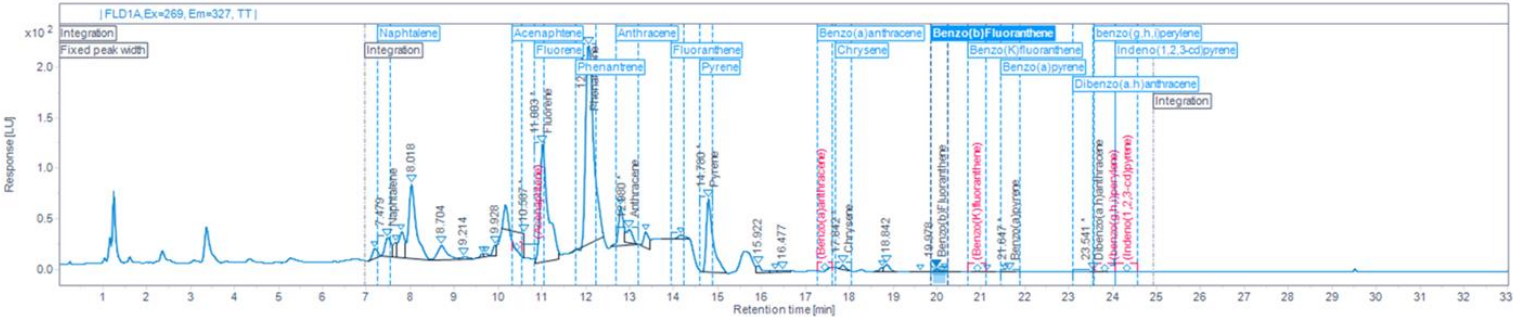
FLD1A,Ex=269, Em=327, TT
Response [LU]
x10 2
Retention time [min]
Integration
Fixed peak width
Naphtalene
Acenaphtene
Fluorene
Phenantrene
Anthracene
Fluoranthene
Pyrene
Benzo(a)anthracene
Chrysene
Benzo(b)Fluoranthene
Benzo(K)fluoranthene
Benzo(a)pyrene
Dibenzo(a,h)anthracene
benzo(g,h,i)perylene
Indeno(1,2,3-cd)pyrene

L9

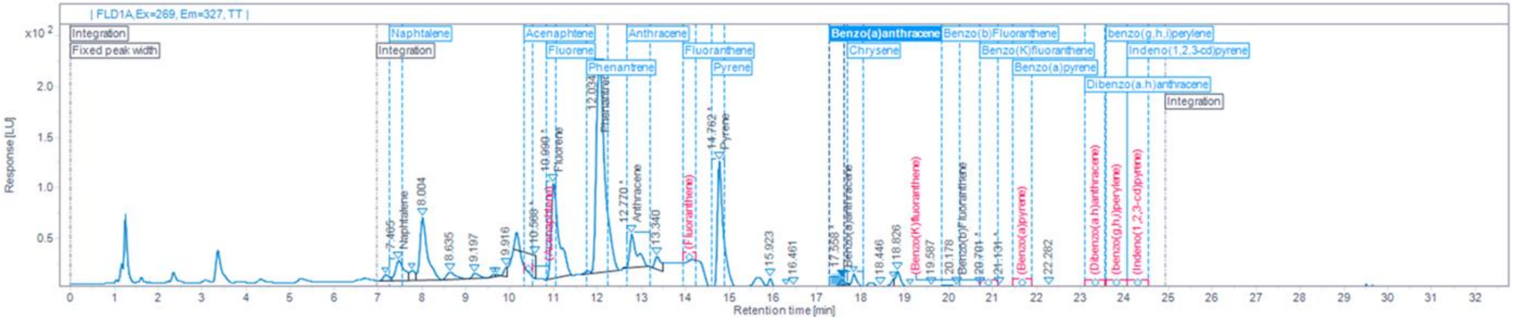
FLD1A,Ex=269, Em=327, TT
Response [LU]
x10 2
Retention time [min]
Integration
Fixed peak width
Naphtalene
Acenaphtene
Fluorene
Phenantrene
Anthracene
Fluoranthene
Pyrene
Benzo(a)anthracene
Chrysene
Benzo(b)Fluoranthene
Benzo(K)fluoranthene
Benzo(a)pyrene
Dibenzo(a,h)anthracene
benzo(g,h,i)perylene
Indeno(1,2,3-cd)pyrene

L10

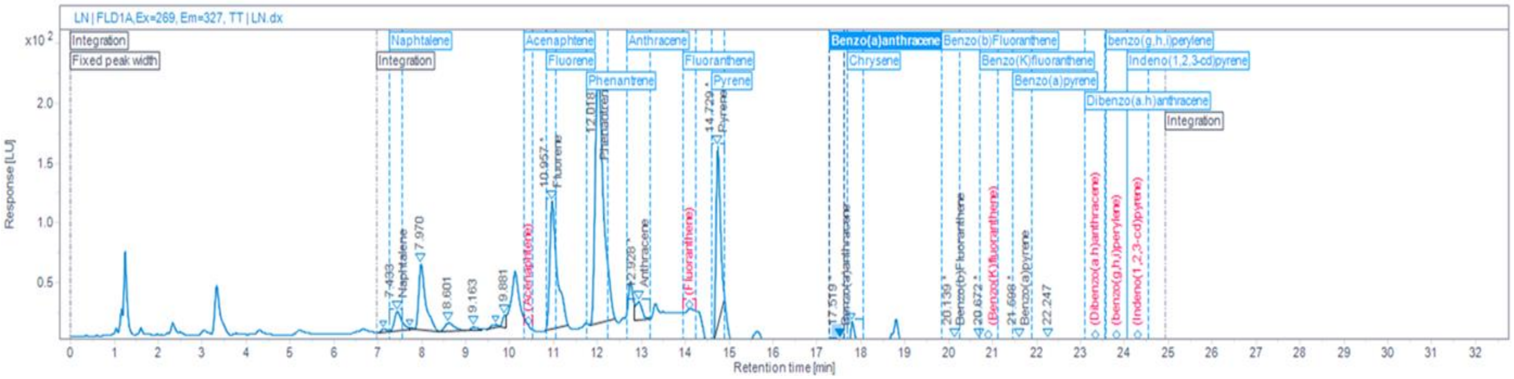

Figure S12. Chromatograms of PAHs in conventional milk samples.

L1B

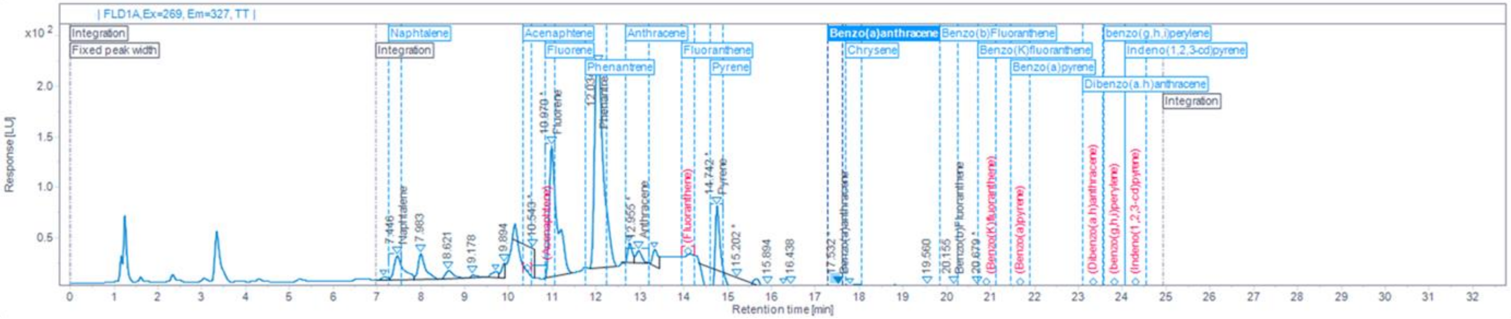

| FLD1A,Ex=269, Em=327, TT |
Response [LU]
Retention time [min]
Naphtalene
Acenaphtene
Fluorene
Phenantrene
Anthracene
Fluoranthene
Pyrene
Benzo(a)anthracene
Chrysene
Benzo(b)Fluoranthene
Benzo(K)fluoranthene
Benzo(a)pyrene
Dibenzo(a,h)anthracene
benzo(g,h,i)perylene
Indeno(1,2,3-cd)pyrene
Integration
Fixed peak width

L6B

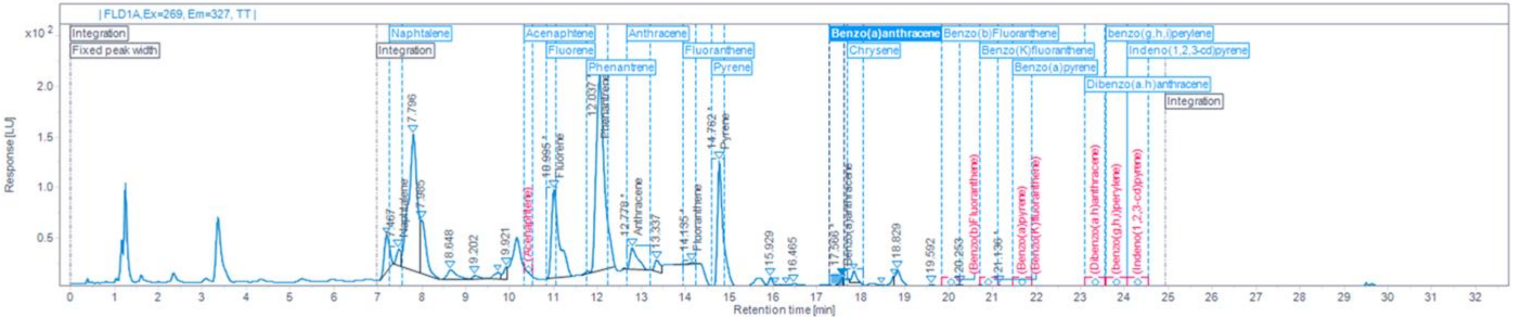

| FLD1A,Ex=269, Em=327, TT |
Response [LU]
Retention time [min]
Naphtalene
Acenaphtene
Fluorene
Phenantrene
Anthracene
Fluoranthene
Pyrene
Benzo(a)anthracene
Chrysene
Benzo(b)Fluoranthene
Benzo(K)fluoranthene
Benzo(a)pyrene
Dibenzo(a,h)anthracene
benzo(g,h,i)perylene
Indeno(1,2,3-cd)pyrene
Integration
Fixed peak width

L7B

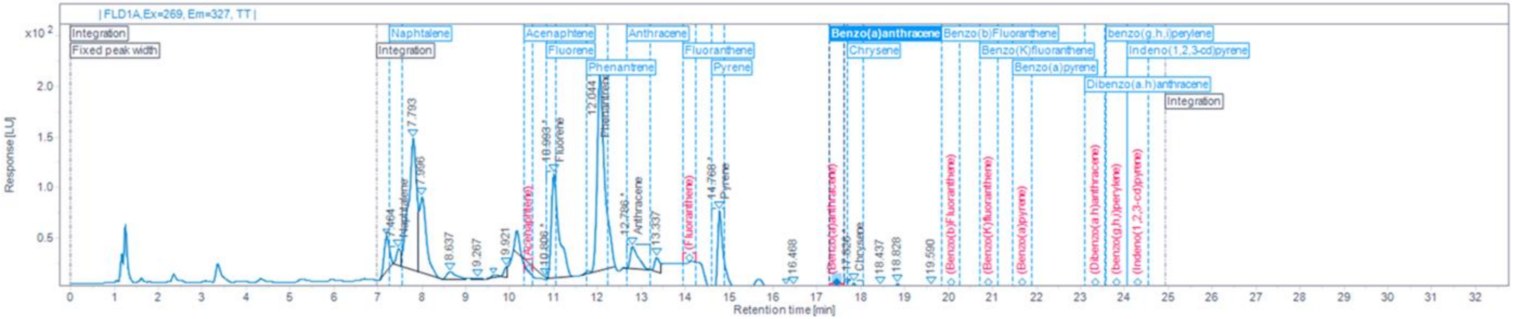

| FLD1A,Ex=269, Em=327, TT |
Response [LU]
Retention time [min]
Naphtalene
Acenaphtene
Fluorene
Phenantrene
Anthracene
Fluoranthene
Pyrene
Benzo(a)anthracene
Chrysene
Benzo(b)Fluoranthene
Benzo(K)fluoranthene
Benzo(a)pyrene
Dibenzo(a,h)anthracene
benzo(g,h,i)perylene
Indeno(1,2,3-cd)pyrene
Integration
Fixed peak width

L8B

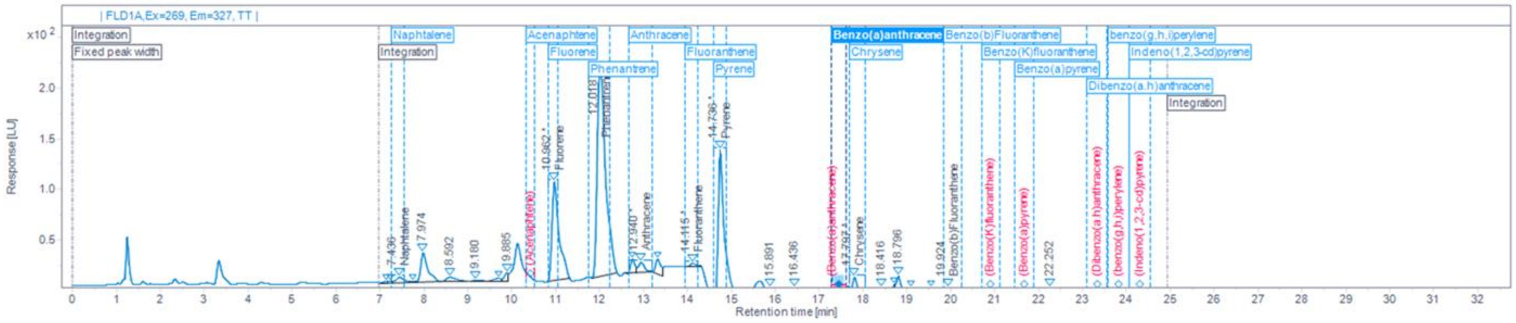

L10B

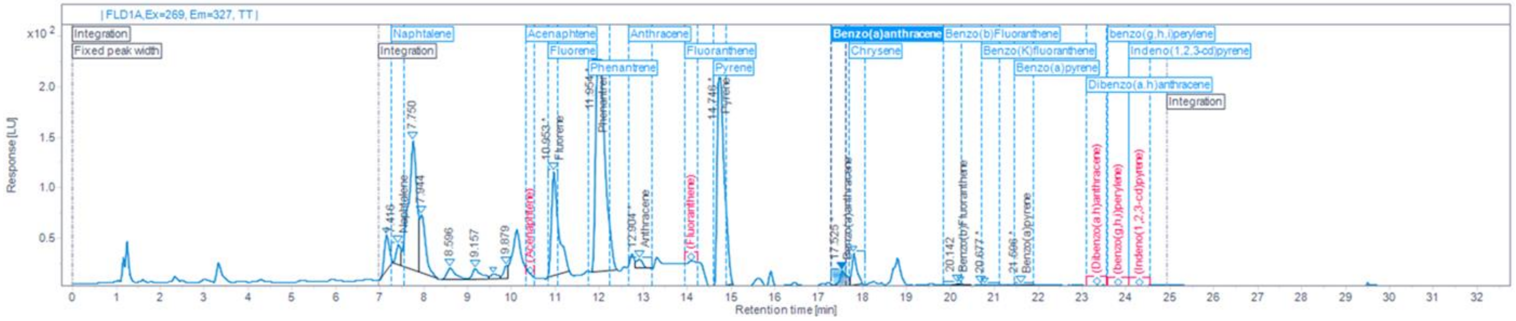

L11B

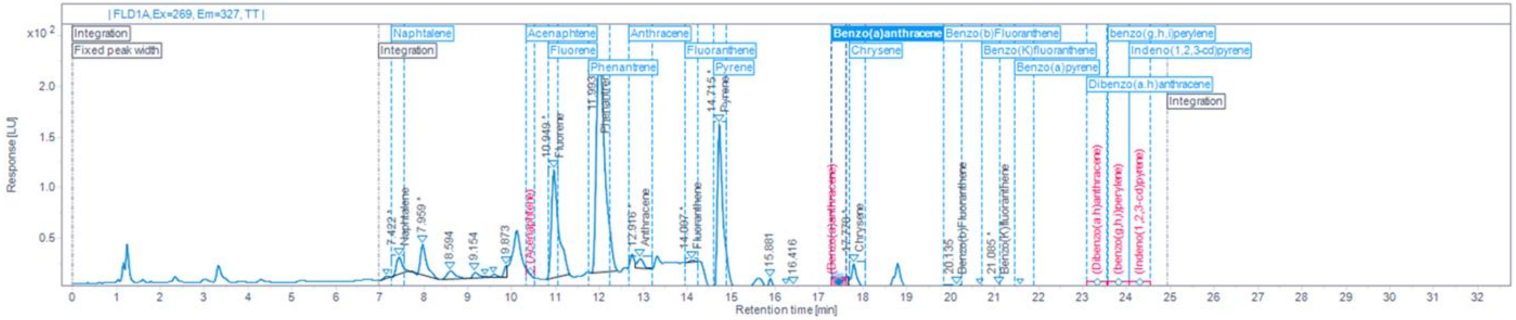

Figure S13. Chromatograms of PAHs in organic milk samples.

Table S6. The concentration of PAHs in conventional and organic milk samples.

Sample code	PAHs* [ng·mL^{-1}]															
	1	2	3	4	5	6	7	8	9	10	11	12	13	14	15	16
Conventional milk																
L1	2.672	*nd*	*nd*	2.577	8.154	0.962	0.369	5.469	0.262	0.661	0.245	*nd*	*nd*	*nd*	*nd*	*nd*
L2	2.140	*nd*	8.772	1.613	7.614	0.440	0.239	2.084	*nd*	0.079	0.071	*nd*	0.004	*nd*	*nd*	*nd*
L3	2.665	*nd*	*nd*	0.925	7.373	1.139	0.328	3.134	0.099	0.253	0.105	*nd*	0.011	*nd*	*nd*	*nd*
L4	2.122	*nd*	*nd*	2.631	7.112	0.954	6.779	*nd*	0.181	0.326	0.104	*nd*	*nd*	*nd*	*nd*	*nd*
L5	nd	*nd*	*nd*	2.519	11.319	0.452	0.641	10.684	1.009	6.441	1.653	0.271	0.049	*nd*	*nd*	*nd*
L6	5.271	*nd*	*nd*	2.368	11.941	1.455	0.268	10.382	1.054	6.162	1.675	*nd*	0.041	*nd*	*nd*	*nd*
L7	2.126	*nd*	*nd*	2.559	6.755	0.252	0.286	2.043	nd	0.114	0.086	0.015	0.005	*nd*	*nd*	*nd*
L8	2.172	*nd*	*nd*	2.982	6.790	0.323	0.413	2.217	nd	0.079	0.172	*nd*	0.008	0.194	*nd*	*nd*
L9	3.328	*nd*	*nd*	2.318	7.643	1.051	*nd*	3.564	0.110	0.348	0.114	*nd*	*nd*	*nd*	*nd*	*nd*
L10	2.413	*nd*	*nd*	2.496	7.424	0.422	*nd*	2.907	0.152	0.513	0.068	*nd*	0.011	*nd*	*nd*	*nd*
Organic milk																
L1B	4.534	*nd*	*nd*	3.278	7.832	0.297	*nd*	2.585	0.031	0.140	0.081	*nd*	*nd*	*nd*	*nd*	*nd*
L6B	1.455	*nd*	*nd*	2.150	6.820	0.707	0.081	3.237	0.099	0.230	*nd*	*nd*	*nd*	*nd*	*nd*	*nd*
L7B	1.916	*nd*	*nd*	2.448	7.862	0.247	0.227	4.384	*nd*	0.643	0.215	0.049	0.010	*nd*	*nd*	*nd*
L8B	0.797	*nd*	*nd*	2.208	7.406	0.259	0.390	3.908	*nd*	0.455	0.048	*nd*	*nd*	*nd*	*nd*	*nd*
L10B	1.829	*nd*	*nd*	2.344	8.513	0.206	*nd*	6.836	0.391	0.713	0.035	0.023	0.004	*nd*	*nd*	*nd*
L11B	1.482	*nd*	*nd*	2.526	6.317	0.731	*nd*	2.320	*nd*	0.195	*nd*	*nd*	*nd*	*nd*	*nd*	*nd*

nd – unidentified

* Legend of compounds: 1-Naphtalene; 2-Acenaphthalene; 3-Acenaphthene; 4-Flourene; 5-Anthracene; 6-Fluoranthene; 7-Pyrene; 8-Benzo[a]anthracene; 9-Chrysene; 10-Benzo[b]fluoranthene; 11-Benzo[k]fluoranthene; 12-Benzo[a]pyrene; 13-Dibenzo[ab]anthracene; 14-Benzo[gbi]perylene; 15-Indeno[1,2,3,-cd]pyrene; 16-Dibenzo[ah]anthracene.

Table S7. The content of heavy metals in milk samples determined by ICP-MS [$mg \cdot L^{-1} \pm SD$].

Sample code	Cr	Mn	Ni	Cu	Zn	Sr	Cd	Pb
L1	0.073±0.001	0.029±0.001	0.013±0.001	0.100±0.001	3.517±0.002	0.345±0.001	<LOD*	0.059±0.001
L2	0.061±0.001	0.020±0.001	0.012±0.001	0.087±0.001	3.069±0.007	0.316±0.001	<LOD*	0.049±0.001
L3	0.047±0.001	0.028±0.001	0.012±0.001	0.099±0.001	3.389±0.007	0.372±0.001	<LOD*	0.016±0.001
L4	0.058±0.001	0.031±0.001	0.021±0.001	0.129±0.001	3.576±0.006	0.404±0.002	0.006±0.001	0.084±0.001
L5	0.094±0.001	0.026±0.001	0.039±0.001	0.135±0.001	4.082±0.007	0.463±0.001	0.011±0.001	0.086±0.001
L6	0.048±0.001	0.029±0.001	0.075±0.001	0.088±0.001	3.633±0.005	0.422±0.002	<LOD*	0.466±0.003
L7	0.051±0.001	0.023±0.001	0.023±0.001	0.081±0.001	3.727±0.007	0.425±0.001	<LOD*	0.188±0.001
L8	0.099±0.001	0.031±0.001	0.052±0.001	0.099±0.001	3.752±0.004	0.438±0.001	<LOD*	0.199±0.002
L9	0.052±0.001	0.023±0.001	0.022±0.001	0.087±0.001	3.881±0.001	0.306±0.001	<LOD*	0.164±0.002
L10	0.050±0.001	0.029±0.001	0.098±0.001	0.071±0.001	3.698±0.009	0.615±0.001	<LOD*	0.311±0.001
L1B	0.048±0.001	0.029±0.001	0.033±0.001	0.071±0.001	3.268±0.003	0.207±0.001	<LOD*	0.052±0.001
L6B	0.099±0.001	0.023±0.001	0.027±0.001	0.142±0.001	3.569±0.003	0.465±0.002	<LOD*	0.051±0.001
L7B	0.081±0.001	0.056±0.001	0.019±0.001	0.114±0.001	3.689±0.006	0.494±0.001	<LOD*	0.049±0.001
L8B	0.054±0.001	0.041±0.001	0.034±0.001	0.126±0.001	3.803±0.006	0.379±0.001	<LOD*	0.071±0.001
L10B	0.097***±0.001	0.027±0.001	0.029±0.001	0.128±0.001	3.497±0.003	0.531±0.001	<LOD*	0.064±0.001
L11B	0.098±0.001	0.021±0.001	0.028±0.001	0.087±0.001	3.722±0.005	0.330±0.001	<LOD*	0.042±0.001

<LOD* – value below the detection limit

Data analysis

Microplastic particles are found in the body of animals, but also of humans in ever-increasing amounts [10,11]. Other research [11-15] has shown that microplastics are frequently found in people's homes, the sources being on the one hand their clothes and shoes, on the other hand, the things used in everyday life. In the urban environment, it has been reported that MPs originate in the air, mainly from the abrasion of car tires, shoes, factories, or from air transport, being thus carried by the wind and in isolated areas, thus depositing on the ground or in water and the end, they are inhaled by people [16,17]. These particles can be inhaled from the air, depositing in the lungs, causing a weakened immune system and various diseases such as infertility, nervous system problems, hearing loss, and even cancer [18-23]. Depending on the location, a person can inhale between 4% and 77% of microplastic particles from the air.

Human exposure to them is a major risk to the health, especially in the case of children (0-3 years). Zang et al. [21] reported in their study that infants have 10 to 20 times more microplastic concentrations in their feces than adults (mainly PET and polycarbonate - PC) and this is very worrying given the fact that children's immunity is extremely low until 14 years old, the brain is in formation until 18 years old. Furthermore, the chemicals added to plastic are causing diseases by their interference with hormones at these early ages. Therefore, based on estimations, it is possible for a human to consume approximately 20,000 microplastic particles per year, and in particular, an adult can ingest up to 5 g of microplastic per week [23,24]. On the other hand, Galloway estimated in her research that the average intake of microplastics through ingestion is 39,000–52,000 particles per person per year [24]. In addition, the presence of MPs fragments has been revealed in the saliva, *i.e.*, 0.33 particles/person [18], colon *i.e.*, 331 particles/person [19], stool *i.e.*, 1-36 particles/ g of stool [11,17] and blood, *i.e.*, 1.6 μg/mL [20] for human, highlighting the exposure to microplastics through ingestion. The hypotheses revealed that microplastics pass through the digestive system without problems and end up in the feces, other MPs was accumulated in different body organs, and others penetrate the cell membranes and end up in the blood [18]. The most worrying studies have shown that they can be transferred from pregnant mothers to developing fetuses through the placenta [25,26].

In the fetus's development stage, as mentioned, as well as after birth, the mothers are becoming involuntary a part of the contamination chain by being exposed daily to a great variety of chemicals/contaminants present in the environment through the most known food, beverages, and personal care products. Therefore, breast milk may get contaminated by these compounds, likely affecting children's health [27,28]. Since the majority of contaminants are lipophilic, they have an increased tendency to deposit in adipose tissue, subsequently; they may be translocated to milk during lactation. [27,29,30]. Not only in the breastfeeding phase but also after, as the usage of feeding bottles and in general, plastic toys, represents an exposure source for infants [31].

One of the contamination causes could be without a doubt the fodder and the water that the farmers choose to use to feed the animals, a process which may affect the quality of the milk implicitly and justifies the presence of microplastics in it. Another possible cause may be the wrapping in the milk processing units. In this regard, the following investigation, related to health risk ingestion or inhalation in terms of MPs and HMs, can be useful for a better understanding the gravity of the phenomenon called pollution throughout the world with serious and irreversible repercussions on the health of children and adults.

References

1. Rathore, C.; Saha, M.; Gupta, P.; Kumar, M.; Naik, A.; de Boer, J. Standardization of micro-FTIR methods and applicability for the detection and identification of microplastics in environmental matrices. *Sci Total Environ.* **2023**, *888*, 164157.
2. Banerjee, A., & Dave, R. N. Validating clusters using the Hopkins statistic. In IEEE International Conference on fuzzy systems (IEEE Cat. No. 04CH37542), Budapest, Hungary, Vol. 1, pp. 149-153, 25-29 July 2004.
3. Yang, J.; Monnot, M.; Sun, Y; Asia, L.; Wong-Wah-Chung, P.; Doumenq, P.; Moulin, P. Microplastics in different water samples (seawater, freshwater, and wastewater): Methodology approach for characterization using micro-FTIR spectroscopy. *Water Reseach.* **2023**, *232*, 119711.
4. Tatli, H.H.; Altuisik, A.; Gedik, K. Microplastic prevalence in Anatolian water frogs (*Pelophylax spp.*). *J. Environ. Manag.* **2022**, *321*, 116029.
5. Altunisik, A. Microplastic pollution and human risk assessment in Turkish bottled natural and mineral waters. *Environ. Sci. Pollut. Res.* **2023**, *30*(14), 39815-39825.
6. Fadare, O.O.; Okffo, E.D.; Olasehinde, E.F. Microparticles and microplastics contamination in African table salts. *Mar. Pollut. Bull.* **2021**, *164*, 112006.
7. Silva, A.B.; Bastos, A.S.; Justino, C.I.L.; da Costa, J.P.; Duarte, A.C.; Rocha-Santos, T.A.P. Microplastics in the environment: Challenges in analytical chemistry - A review. *Anal. Chim. Acta*. **2018**, *1017*, 1–19.
8. Altunisik, A. Prevalence of microplastics in commercially sold soft drinks and human risk assessment. *J. Environ. Manag.* **2023**, *336*, 117720.
9. Garside, P.; Wyeth, P. Identification of Cellulosic Fibres by FTIR Spectroscopy I: Thread and Single Fibre Analysis by Attenuated Total Reflectance. *Stud. Conserv.* **2003**, *48*(4), 269-275.
10. Ali, M.; Xu, D.; Yang, X.; Hu, J. Microplastics and PAHs mixed contamination: An in-depth review on the sources, co-occurrence, and fate in marine ecosystems. *Water Research.* **2024**, 257, 121622.
11. Li, Y.; Chen, L.; Zhou, N.; Chen, Y.; Ling, Z.; Xiang, P. Microplastics in the human body: A comprehensive review of exposure, distribution, migration mechanisms, and toxicity. *Sci. Total Environ.* **2024**, *946*, 174215.
12. Abbasi, S.; Turner, A. Human exposure to microplastics: a study in Iran. *J. Hazard. Mater.* **2021**, *403*, 123799.
13. Lee, J.; Jeong, S. Approach to an answer to "How dangerous microplastics are to the human body": A systematic review of the quantification of MPs and simultaneously exposed chemicals. *J Hazard Mater.* **2023**, *460*, 132404.
14. Kim, M.J.; Kim, J.A.; Song, J.A.; Kho, K.H.; Choi, C.Y. Synthetic microfiber exposure negatively affects reproductive parameters in male medaka (Oryzias latipes). *Gen. Comp. Endocrinol.* **2023**, *334*, 114216.
15. Zhai, X.; Zheng, H.; Xu, Y.; Zhao, R.; Wang, W.; Guo, H. Characterization and quantification of microplastics in indoor environments. *Helyion.* **2023**, *9*(5), e15901.
16. Dris, R.; Gasperi, J.; Saad, M.; Mirande, C.; Tassin, B. Synthetic fibers in atmospheric fallout: A source of microplastics in the environment?. *Mar. Poll. Bull.* **2016**, *104*(1–2), 290–293.
17. Schwabl, P.; Koppel, S.; Konigshofer, P.; Bucsics, T.; Trauner, M.; Reiberger, T.; Liebmann, B. Detection of various microplastics in human stool. *Ann. Intern. Med.* **2019**, *171*(7), 453–458.
18. Zhang, Y.; Kang, S.; Allen, S.; Allen, D.; Gao, T.; Sillanpa, M. Atmospheric microplastics: a review on current status and perspectives. *Earth Sci. Rev.* **2020**, *203*, 103118.
19. Ibrahim, Y.S.; Anuar, S.T.; Azmi, A.A.; Khalik, W.M.A.W.M.; Lehata, S.; Hamzah, S.R.; Ismail, D.; Ma, Z.F.; Dzulkarnaen, A.; Zakaria, Z.; Mustaffa, N.; Sharif, S.E.T.; Lee, Y.Y. Detection of microplastics in human colectomy specimens. *JGH Open.* **2020**, *5*(1), 116–121.
20. Leslie, H.A.; van Velzen, M.J.M.; Brandsma, S.H.; Vethaak, A.D.; Garcia-Vallejo, J.J.; Lamoree, M.H. Discovery and quantification of plastic particle pollution in human blood. *Environ. Int.* **2022**, *163*, 107199.
21. Zhang, J.; Wang, L.; Kannan, K. Polyethylene terephthalate and polycarbonate microplastics in pet food and feces from the United States. *Environ. Sci. Technol.* **2019**, *53*(20), 12035-12042.
22. Wong, S.L.; Nyakuma, B.B.; Wong, K.Y.; Lee, C.T.; Lee, T.H.; Lee, C.H. Microplastics and nanoplastics in global food webs: a bibliometric analysis (2009-2019). *Mar. Pollut. Bull.* **2020**, *158*, 111432.
23. Lwanga, E.H.; Vega, J.M.; Quej, V.K.; Chi, J.A.; Cid, L.S.; Chi, C.; Segura, G.E.; Gertsen, H.; Salanki, T.; Ploeg, M.; Koelmans, A.A.; Geissen, V. Field evidence for transfer of plastic debris along a terrestrial food chain. *Sci. Rep.* **2017**, *7*, 14071.
24. Galloway, T.S. Micro- and nano-plastic and human health. In Marine Anthropogenic Litter; Bergmann, M., Gutow, L., Klages, M., Eds.; Springer, Cham, Germany, 2015; pp. 343–366.
25. Ragusa, A.; Svelato, A.; Santacroce, C.; Catalano, P.; Notarstefano, V.; Carnevali, O.; Papa, F.; Rongioletti, M.C.A.; Baiocco, F.; Draghi, S.; D'Amore, E.; Rinaldo, D.; Matta, M.; Giorgini, E. Plasticenta: First evidence of microplastics in human placenta. *Environ. Int.* **2021**, *146*, 106274.
26. Zhu, L.; Zhu, J.; Zuo, R.; Xu, Q.; Qian, Y.; An, L. Identification of microplastics in human placenta using laser direct infrared spectroscopy. *Sci. Total Environ.* **2023**, *856*(1), 159060.

27. Ragusa, A.; Notarstefano, V.; Svelato, A.; Belloni, A.; Gioacchini, G.; Blondeel, C.; Zucchelli, E.; De Luca, C.; D'Avino, S.; Gulotta, A.; Carnevali, O.; Giorgini, E. Raman Microspectroscopy Detection and Characterisation of Microplastics in Human Breastmilk. *Polym.* **2022**, *14*(13), 2700.
28. Karthikeyan, B.S.; Ravichandran, J.; Aparna, S.R.; Samal, A. ExHuMId: A curated resource and analysis of Exposome of Human Milk across India. *Chemosphere*. **2021**, *271*, 129583.
29. Mead, M.N. Contaminants in Human Milk: Weighing the Risks against the Benefits of Breastfeeding. *Environ. Health Perspect.* **2008**, *116*, A426–A434.
30. Vasios, G.; Kosmidi, A.; Kalantzi, O.I.; Tsantili-Kakoulidou, A.; Kavantzas, N.; Theocharis, S.; Giaginis, C. Simple physicochemical properties related with lipophilicity, polarity, molecular size and ionization status exert significant impact on the transfer of drugs and chemicals into human breast milk. *Expert Opin. Drug Metab. Toxicol.* **2016**, *12*, 1273–1278.
31. Liu, S.; Guo, J.; Liu, X.; Yang, R.; Wang, H.; Sun, Y.; Chen, B.; Dong, R. Detection of various microplastics in placentas, meconium, infant feces, breastmilk and infant formula: A pilot prospective study. *Sci. Total Environ.* **2023**, *854*, 158699.
